# Seeking safety intervention for comorbid post‐traumatic stress and substance use disorder: A meta‐analysis

**DOI:** 10.1002/brb3.2999

**Published:** 2023-04-10

**Authors:** Athena D. F. Sherman, Monique Balthazar, Wenhui Zhang, Sarah Febres‐Cordero, Kristen D. Clark, Meredith Klepper, Mercy Coleman, Ursula Kelly

**Affiliations:** ^1^ The Nell Hodgson Woodruff School of Nursing Emory University Atlanta Georgia; ^2^ Byrdine F. Lewis College of Nursing and Health Professions Georgia State University Atlanta Georgia; ^3^ School of Nursing University of California San Francisco San Francisco California; ^4^ Johns Hopkins University School of Nursing Johns Hopkins University Baltimore Maryland; ^5^ Atlanta VA Health Care System Atlanta Georgia

**Keywords:** behavioral therapy, coping, mental health, PTSD

## Abstract

**Problem statement:**

Seeking Safety (SS) is a widely implemented cognitive‐behavioral therapy for comorbid post‐traumatic stress disorder (PTSD) and substance use disorder (SUD). It is a present‐focused coping skills model that is highly flexible, with varied methods of delivery, to maximize acceptability and client access. The purpose of this meta‐analysis is to examine the effect of SS on comorbid PTSD and SUD across randomized control trials (RCTs). In addition, ours is the first meta‐analysis to examine the dose‐response of SS by comparing delivery of all 25 SS topics versus fewer.

**Methods and design:**

Articles published before January 2, 2023 (CINAHL *n* = 16, PsycINFO *n* = 31, MEDLINE *n* = 27, Cochrane *n* = 38, and Scopus *n* = 618) were searched. Seven studies were included for meta‐analysis and dose‐response analysis.

**Results:**

Based on effect sizes (ES), meta‐analysis revealed that SS has a medium group, time (*p* = .04), and time by group effect on substance use per the Addiction Severity Index at 3 months and a small effect on Clinician‐Administered PTSD Scale scores by group, a large effect by time, and a medium time by group (*p* = .002) effect at 6 months. Based on the pooled ES examining various measures across multiple timepoints, SS had small to medium effects on substance use by time, group, or time by group and medium to large effects on PTSD symptoms by time, group, or time by group (except for the group effect at 3‐month follow‐up). Significant effects were found for substance use by time at 3 and 6 months and for PTSD postintervention, at 6 months and 9 months by group, time, and time by group while only by time at 3 months. Meta‐regression revealed that partial dose versions of SS generally function as well as the full dose version of SS when observing long‐term effects (greater than 3 months).

**Discussion:**

Findings suggest SS has merit in treating PTSD symptoms and SUD. Based on the summarized effect sizes, SS appears more effective in reducing PTSD than substance use, which converges with the larger treatment outcome literature that consistently finds this. We explore reasons that treatment of SUD is more challenging than treating PTSD and offer suggestions for practitioners. We emphasize the need for future studies to utilize common measures and provide full details of treatment delivery for optimal comparison across studies.

## Graphical abstract

The purpose of this meta‐analysis is to examine the effect of Seeking Safety (SS on PTSD and SUD across randomized control trials (RCTs) and examine the dos response. Findings suggest SS has merit in treating PTSD symptoms and substance use ‐ SS appears more effective in reducing PTSD than substance use, which converges with the larger treatment outcome literature that consistently finds this. We explore reasons why treatment of SUD is more challenging than treating PTSD and offer suggestions for practitioners.

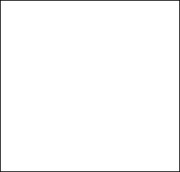

## 1 INTRODUCTION

The treatment of post‐traumatic stress disorder (PTSD) and comorbid substance use disorder (SUD) is costly and highly challenging for clinicians. Patients with comorbid PTSD symptoms and SUD are less able to process trauma and regulate their emotions than people with only one disorder or the other (Kaysen et al., 2011). Approximately 46% of people with PTSD have comorbid substance use (Pietrzak et al., 2011). Preexisting SUD increases the risk for PTSD, and those with PTSD are more likely to use substances, at times resulting in SUD (Pietrzak et al., 2011; van den Berk‐Clark & Patterson Silver Wolf, 2017). Comorbid PTSD and SUD are particularly debilitating and have a poor prognosis for quality of life (Kessler et al., 2009; McCauley et al., 2012; Najavits et al., 1997; Pietrzak et al., 2011; Vujanovic et al., 2018). Those with comorbid PTSD symptoms and substance use experience less treatment adherence and response, worse chronic physical health problems, higher rates of suicide attempts, and poorer social functioning compared to people with either PTSD symptoms or SUD (McCauley et al., 2012; Najavits et al., 1997; Roberts et al., 2015). Concurrent and effective treatment of PTSD and comorbid SUD is necessary to reduce symptom severity, decrease rates of chronic PTSD, and reduce risk of suicide (Forehand et al., 2019; Najavits et al., 2020; van den Berk‐Clark & Patterson Silver Wolf, 2017).

Seeking Safety is a widely implemented, manualized cognitive‐behavioral therapy for PTSD and/or SUD, designed for individual or group modality. It is a present‐focused coping skills model that is highly flexible to maximize acceptability and client access (e.g., session length, pacing, and order of topics can vary; Najavits, 2002; Najavits et al., 1998). It is also notable for being able to be delivered by peers and paraprofessionals, in addition to professionals. SS sessions are structured, starting with a check‐in (each person states how they are feeling, what good coping they have done, any unsafe behavior, whether they completed their commitment aka homework from the prior session, and an update on community resources they have engaged with since the last session). After that there is an inspiring quotation, followed by exploration of handouts for that day's topic, and finally a check‐out (each person states one thing they got from the session, any problems with the session, and a community resource they will contact if needed; Najavits, 2002).

SS offers 25 topics based on five key principles: (1) **Safety** as the overarching goal (helping patients attain safety in their relationships, thinking, behavior, and emotions). (2) **Integrated** treatment (working on both trauma and substance abuse at the same time if the person has both). (3) **A focus on ideals** to counter the loss of ideals in both PTSD and SUD. (4) **Four content areas**: cognitive, behavioral, interpersonal, case management. (5) **Attention to clinician processes** (clinicians' emotional responses, self‐care, etc.)

The 25 topics can be conducted in any order and as few or many as time allows: Introduction/Case Management, Safety, PTSD: Taking Back Your Power, When Substances Control You, Honesty, Asking for Help, Setting Boundaries in Relationships, Getting Others to Support Your Recovery, Healthy Relationships, Community Resources,  Compassion, Creating Meaning, Discovery, Integrating the Split Self, Recovery Thinking, Taking Good Care of Yourself, Commitment, Respecting Your Time, Coping with Triggers, Self‐Nurturing, Red and Green Flags, Detaching from Emotional Pain (Grounding). Life Choices, and Termination.

SS has undergone efficacy and effectiveness trials in many variations and by many independent investigators, including (a) individual delivery (e.g., Hien et al., 2004), group delivery (e.g., Boden et al., 2012; Crisanti et al., 2019) (b) integration with pharmacotherapy (Hien et al., 2015) or other treatment modalities (e.g., Murphy et al., 2019; Ragg et al., 2019), (c) delivery by peers and case managers as well as clinicians (Crisanti et al., 2019; Desai et al., 2008), and (d) examination in non‐healthcare settings (e.g., community‐based, jail/prison‐based), with a broad range of racially and ethnically diverse participants. Samples in studies of SS included veterans (Boden et al., 2012; Desai et al., 2008), people with physical disabilities (Anderson & Najavits, 2014), homeless (Desai et al., 2008), incarcerated women and men (Lynch et al., 2012; Zlotnick et al., 2009; Barrett et al., 2015), adolescent girls (Najavits et al., 2006), indigenous populations (Marsh, 2016), pregnant women (Shenai et al., 2019), and transgender women (Empson et al., 2017; Takahashi, 2020).

Given that SS was designed for use in whatever timeframe and number of sessions and topics is desired by the clinician, SS has been tested at times in various ways. Some studies used all 25 topics, typically conducted as one topic per session; while other studies used 6 to 12 topics, delivered once per session (e.g., Hien et al., 2012, 2009; Ghee et al., 2009a,b). A 2013 comprehensive literature review on all treatments for PTSD and SUD indicated that SS studies that used fewer topics obtained may have more mixed results than those that used all 25. That review also indicated that SS was the most rigorously studied treatment for comorbid PTSD and SUD; and that “most models had more effect on PTSD than SUD, suggesting that SUD is harder to treat” (p. 433, Najavits & Hien, 2013).

In 2016, a meta‐analysis by Lenz et al. (2016) confirmed SS as an effective, evidence‐based treatment for treating comorbid PTSD (medium effect) and substance use (modest effect) symptoms (Lenz, 2016). By the end of 2020 the literature evaluating SS nearly doubled (Seeking Safety, 2020) compared to Lenz et al. However, thus far, no meta‐analysis has examined the dose‐response of treatment effects, that is, how the number of components covered may affect treatment outcomes. Therefore, our meta‐analysis aims to examine the effect of SS on PTSD and SUD across randomized control trials (RCTs; time, group, and time × group comparisons) and examine the dose‐response of SS by comparing the effects of the full version to the abbreviated versions of SS.

## 2 METHODS

### 2.1 Search strategy and selection of articles

Articles published prior to January 2, 2023, were identified from five databases (CINAHL (*n* = 16), PsycINFO (*n* = 31), MEDLINE (*n* = 27), Cochrane (*n* = 38), and Scopus (*n* = 618)]) for systematic review and meta‐analysis. Search terms comprised four categories: (1) “Seeking Safety,” (2) PTSD (PTSD OR posttraumatic OR post‐traumatic OR post OR traum^*^ OR stress), (3) substance use (“Substance ^*^use” OR substance OR drug^*^ OR narcotics OR pharma^*^ OR medica^*^), and (4) RCT (random^*^ AND control^*^). Additional filters were used to limit the search to articles published in English and to studies with human participants. Eligible articles for meta‐analysis met the following inclusion criteria: (1) studies of efficacy or effectiveness of SS among adult participants; (2) must report both PTSD and SUD outcomes at baseline and one additional timepoint; (3) RCT; (4) published in English, (5) there was information on sample sizes to calculate standard errors of the standardized mean differences (SMDs); (6) were published in a peer‐reviewed journal. Articles were excluded if they did not meet the following criteria for meta‐analysis: the means and standard deviations of intervention and control groups of the outcomes (PTSD or substance use) were reported in at least three studies at a common timepoint (e.g., 3, 6, or 9 months postintervention; Basu, 2017). Studies were excluded from dose‐response analysis if they did not state how many topics of SS were delivered. Covidence systematic review software (Veritas Health Innovation, 2021) was used to manage article selection. This systematic review was exempt from institutional review board (IRB) review at Emory University.

Data Extraction Data from each included article were extracted using double entry by two independent reviewers to ensure accuracy. Discrepancies were resolved via a third reviewer. Extracted data included (1) demographic information on study participants; (2) details of intervention design and delivery; and (3) point estimate and measure of dispersion (e.g., standard error or 95% confidence interval) reflecting the effect of intervention on PTSD symptoms and substance use.

### 2.2 Analysis

All retained articles were described briefly in tables and underwent quality appraisal and meta‐analyses. The effects of the interventions under study were evaluated in relation to two outcomes of interest (PTSD symptoms and substance use). Each outcome was evaluated using three comparisons: (1) difference at baseline before and after the intervention within the intervention group (denoted as “Time” and measured at follow‐ups); (2) difference between the intervention and the control group at follow‐ups (denoted as “Group”); and (3) difference‐of‐differences whereby pre‐ vs. postintervention changes at follow‐ups were compared in the intervention and the control groups (denoted as “Time by Group”). For each comparison, R meta package (Schwarzer, 2020) was used to generate pooled SMD or effect sizes (ES) for Time, Group, and Time by Group comparisons (Lenhard & Lenhard, 2016). For studies that measured the outcomes using the same scales, SMD were calculated by Cohen's *d* (Cohen, 2013). For studies that measured the outcomes using different scales, SMD were calculated by Hedges’ *g* (Hedges, 1981). Stratified meta‐analyses calculated the pooled SMD by group of studies that used different versions of the SS intervention (full, abbreviated, and all studies). SMD and ES estimates were interpreted as “small” (< 0.20), “medium” (0.20–0.80) and “large” (>0.80) according to convention (Cohen, 2013). The result of each meta‐analysis was expressed as the pooled SMD or meta‐ES accompanied by a corresponding 95% confidence interval (CI) and a prediction interval that presents the expected range of true effects across similar studies (IntHout et al., 2016). All meta‐analyses were performed using random effects models. Heterogeneity of results across studies was assessed by the *I* ^2^ and *τ* ^2^ statistics and a *Q* test. Based on the meta‐analysis results, meta‐regressions further explored if the version of the intervention (full or abbreviated version of SS) was associated with the time, group, and time by group effects for studies that measured the outcomes using different scales at different follow‐ups. The significance level alpha was set to 0.05.

### 2.3 Assessment of bias in individual studies

Two researchers independently assessed each included article for risk of bias using the Revised Cochrane Risk‐of‐Bias Tool for Randomized Control Trials (RoB2) (Sterne et al., 2019). This instrument assesses five domains: randomization process, deviations from intended interventions, missing outcome data, measurement of the outcome, and selection of the reported results. Studies were then characterized as having “high risk of bias” (i.e., one or more domains were deemed to be high risk or concern regarding multiple domains), “some concerns” (i.e., one or more domains were deemed to convey some concerns, but no high risk in any domains), or “low risk of bias” (i.e., all domains conveyed low risk). Where the two researchers disagreed, discrepancies were resolved by consensus.

## 3 RESULTS

### 3.1 Description of reviewed studies

**FIGURE 1:**
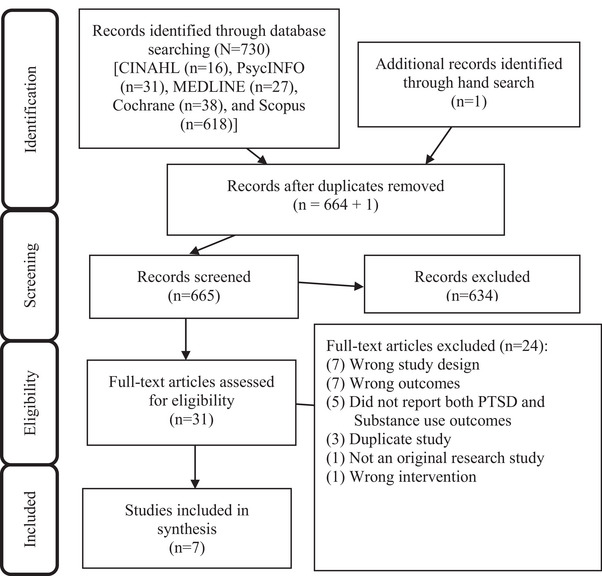
The Preferred Reporting Items for Systematic Reviews and Meta‐Analyses (PRISMA) diagram.

In total, 730 articles were identified from the initial search. One additional article (not indexed in the reviewed databases) was identified from a hand search of the reference lists. Duplicates were removed, yielding 665 articles for screening. Figure 1 shows the Preferred Reporting Items for Systematic Reviews and Meta‐Analyses (PRISMA) flow diagram (Page et al., 2021). Screening of titles and abstracts resulted in exclusion of 634 articles. The remaining 31 articles received a full‐text review, and 7 were included in the study. The final seven articles were retained for data extraction and meta‐analysis (Boden et al., 2012; Ghee et al., 2009; Hien et al., 2004, 2009; Najavits et al., 2018; Schafer et al., 2019; Zlotnick et al., 2009).

The seven RCTs were assessed by RoB2 tool (Sterne et al., 2019) and all were determined to be “low risk of bias.” All seven studies were conducted in the United States and published in peer‐reviewed journals between 2004 and 2019, two of which were published after the completion of the most recent meta‐analysis of SS outcomes (Lenz, 2016; see Table 1 for details). Five studies were recruited from community outpatient substance use services, one was recruited from a minimum‐security prison substance use treatment program, and the final study was recruited from a Veterans Affairs substance use clinic. Three of the seven studies evaluated the full version of SS. The remaining four studies evaluated abbreviated versions of SS, with protocols ranging from 6 to 17 sessions (topics covered are reported in Table 1) with durations of 60–90 min; two of these included SS plus treatment as usual (TAU). Sample settings included outpatient clinics in the VA and the community, community residential substance use treatment facility and a prison. Table 2 includes a list of the measures and key findings of the seven included studies.

**TABLE 1:** Description of included studies

**Source**	**Participants and setting**	**Age *M* (*SD*)**	**Sex**	**Experimental group**	**Control or comparison**
Boden et al. (2012)	Military veterans recruited from a Veterans Affairs outpatient SUD clinic	54.0 (9.6)	100% male	SS full version, twice‐weekly group sessions + weekly individual case management sessions (*N* = 49)	TAU^a^ group therapy (*N* = 49)
Cash Ghee et al. (2009a,b)	Adults in community residential SUD treatment facility	34.7 (8.7)	100% female	SS condensed to six twice‐weekly 90‐min group sessions + TAU (*N* = 36) **Topics covered**: Introduction to Safety, PTSD: Taking Back Your Power, Detaching from Emotional Pain (Grounding), Setting Boundaries in Relationships, Asking for Help, and Commitment	TAU (*N* = 52)
Hien et al. (2004)	Outpatients recruited through SUD treatment programs and community advertisements	37.3 (6.3)^b^	100% female	SS full version, twice‐weekly 1‐h individual sessions (*N* = 41)	1. Relapse prevention individual therapy (*N* = 34) 2. Community care (*N* = 32^c^)
Hien et al. (2009)	Outpatients recruited through SUD treatment programs and community advertisements	39.2 (9.3)	100% female	SS condensed to 12 75‐ to 90‐min group sessions (*N* = 176) **Topics covered**: safety, taking back power from PTSD, when substances are in control, honesty, setting boundaries in relationships, compassion, healing from anger, creating meaning, integrating the split self, taking good care of oneself, red and green flags, and detaching from emotional pain (grounding).	Women's Health Education (*N* = 177)
Najavits et al. (2018)	Outpatients recruited via clinicians, flyers, and word‐of‐mouth	48.75 (10.8)	73.1% male 26.9% female	SS condensed to 17 weekly 1‐h individual sessions (*N* = 26) **Topics covered**: not reported	CC (*N* = 26)
Schäfer et al. (2019)	Outpatients recruited via clinicians, flyers, and word‐of‐mouth	40.9 (11.4)	100% female	SS condensed to 16 weekly 90‐min group sessions + TAU (*N* = 115) **Topics Covered**: introduction/case management, detaching from emotional pain (grounding), safety, when substances control you, red and green flags, asking for help, setting boundaries in relationships, self‐nurturing, PTSD: taking back your power, commitment, recovery thinking, coping with triggers, honesty, integrating the split self, healing from anger and termination	1. Relapse Prevention Training (modified) group therapy + TAU (*N* = 111) 2. TAU (*N* = 117)
Zlotnick et al. (2009)	Prisoners in a minimum‐security prison recruited from a voluntary in‐prison residential SUD treatment program	34.6 (7.4)	100% female	SS full version, 90‐min group sessions three times a week + TAU (*N* = 23)	TAU (*N* = 21)

^a^ TAU groups included patients with and without PTSD symptomatology.

^b^ Calculated from the reported mean ages for each of the 3 groups.

^c^ Nonrandomized nonspecific comparison group.

CC, Creating Change; *M*, mean; PTSD, post‐traumatic stress disorder; *SD*, standard deviation; SS, Seeking Safety, a cognitive‐behavioral integrated treatment for PTSD and SUD; SUD, substance use disorder; TAU, treatment as usual.

**TABLE 2:** Measures and key findings of included studies

**Source**	**Key outcomes** **examined**	**Measures used**	**Key findings**
Boden et al. (2012)	PTSD severity	Impact of Events Scale—Revised	SS was associated with significantly greater improvements in drug use over time as compared to TAU. SS performed as well as TAU in reducing alcohol use and PTSD symptom severity.
	Drug or alcohol use severity	Addiction Severity Index	
Cash Ghee et al. (2009a,b)	Sexual‐abuse‐related trauma symptom severity	Trauma Symptoms Checklist: Sexual Abuse Trauma Index	Condensed SS produced lower sexual abuse trauma symptoms at the 30‐day posttreatment assessment compared to TAU. No statistical evidence for overall PTSD symptom severity reduction. Condensed SS intervention resulted in higher drug relapse rates compared to TAU.
	Overall trauma severity	Modified PTSD Symptom Scale	
	Drug abstinence status	InstaCup Drug Screen	
Hien et al. (2004)	PTSD severity	Clinician‐Administered PTSD Scale‐1; Revised Impact of Event Scale; Clinical Global Impression of PTSD	SS and RP resulted in a significant reduction in substance use and PTSD symptom severity. CC participants showed no significant changes; in the case of PTSD, their symptoms worsened over time.
	Substance use severity	Substance Use Inventory; Clinical Global Impression; Structured Clinical Interview; Urine Screens	
	Psychiatric Symptom Severity	Clinical Global Impressions; The Global Assessment Scale; Hamilton Depression Rating Scale	
Hien et al. (2009)	PTSD severity	Clinician‐Administered PTSD Scale‐1; The Post Traumatic Stress Disorder Symptom Scale—Self Report	SS and WHE showed large clinically significant reductions in CAPS‐1 and PSS‐SR but no reliable difference between the two conditions. Substance use outcomes were not significantly different over time between conditions and at follow‐up showed no significant change from baseline. Study results do not favor SS over WHE as an adjunct to substance use disorder treatment for women with PTSD.
	Alcohol Use	Saliva Alcohol Tests; Substance Use Inventory	
	Substance abstinence rate	Urine Drug Screen; Composite International Diagnostic Interview; The Substance Use Inventory	
Najavits et al. (2018)	PTSD severity	Mini Neuropsychiatric Interview—PTSD Module; Trauma History Questionnaire; PTSD Checklist; Trauma Related Guilt Inventory; World Assumptions Scale	All of the primary and secondary outcomes improved overtime with no difference between conditions.
	Alcohol use severity	Breathalyzer; Mini Neuropsychiatric Interview—Alcohol Use Disorder; Addiction Severity Index‐Lite—Alcohol Composite	
	Drug use severity	Urinalysis; Mini Neuropsychiatric Interview—Substance Use Disorder; Addiction Severity Index‐Lite—Drug Composite; Beliefs about Substance Use; Nicotine Screen	
Schäfer et al. (2019)	PTSD severity	PTSD Symptom Scale Interview; Posttraumatic Diagnostic Scale	There were similar decreases in PTSD symptom severity among the 3 conditions (Seeking Safety, Relapse Prevention, and Treatment as Usual). SS+TAU showed superior efficacy to TAU along and equal efficacy to RPT + TAU on depression and emotion regulation. RPT + TAU was more effective than TAU alone and as effective as SS + TAU on number of substance‐free days and alcohol severity, but not drug severity.
	SUD severity	Addiction Severity Index‐Lite	
	Depression	Beck Depression Inventory II	
	Emotion dysregulation	Difficulties in Emotion Regulation Scale	
Zlotnick et al. (2009)	PTSD diagnosis and severity	Clinician‐Administered Posttraumatic Stress Disorder Scale‐I; Trauma Symptom Checklist 40; Trauma History Questionnaire	Both conditions (SS + TAU and TAU alone) in showed significant improvements from intake to subsequent time points on measures of each of the key domains (e.g., PTSD symptom severity, substance use, psychopathology, legal problems), however, there were no significant differences between conditions on any measure in the primary analyses. Secondary analyses (i.e., paired *t*‐tests/chi squares) suggest some benefit for SS above TAU, most notably on measures of psychopathology.
	Substance use	Structured Clinical Interview for DSM‐IV—Patient Version; Addiction Severity Index; The Time Line Follow Back;	
	Prison recidivism	Self‐report; Prison census	
	Legal problems	Legal composite score of the Addiction Severity Index	
	Psychopathology	Brief Symptom Inventory	

CC, Creating Change; PTSD, post‐traumatic stress disorder; SS, Seeking Safety, a cognitive‐behavioral integrated treatment for PTSD and SUD; RP, relapse prevention; RPT, relapse prevention training;SUD, substance use disorder; TAU, treatment as usual; WHE, women's health education.

### 3.2 Meta‐analysis

**FIGURE 2:**
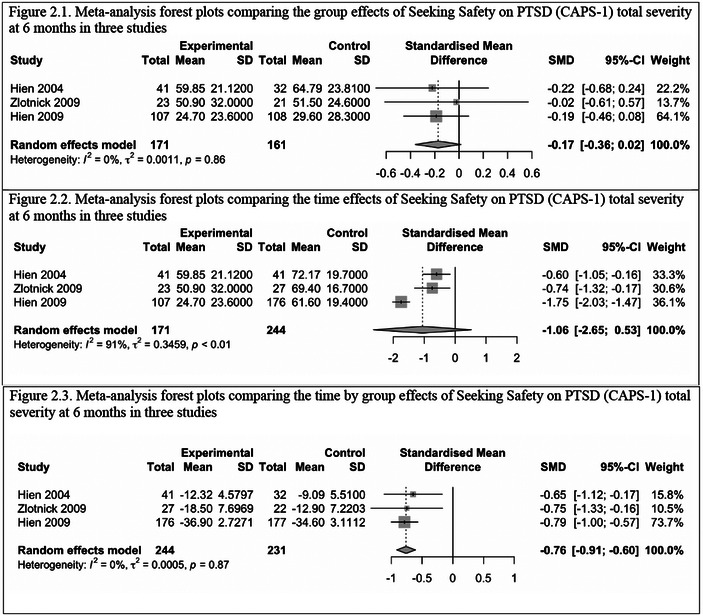
Meta‐analysis forest plots comparing the group, time, and time by group effects of Seeking Safety on PTSD (CAPS‐1) total severity at 6 months in three studies. (1) Meta‐analysis forest plots comparing the group effects of Seeking Safety on PTSD (CAPS‐1) total severity at 6 months in three studies. (2) Meta‐analysis forest plots comparing the time effects of Seeking Safety on PTSD (CAPS‐1) total severity at 6 months in three studies.

**FIGURE 3:**
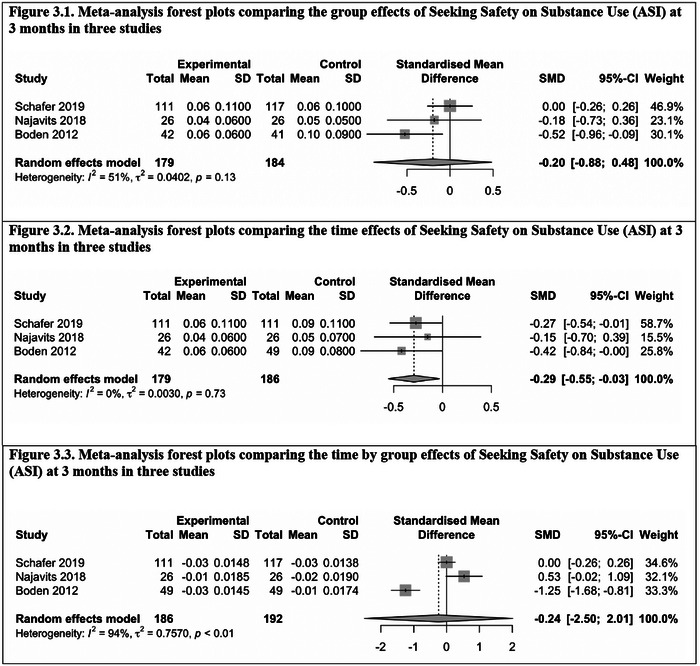
Meta‐analysis forest plots comparing the group, time, and time by group effects of Seeking Safety on Substance Use (ASI) at 3 months in three studies. **(1) Meta‐analysis forest plots comparing the group effects of Seeking Safety on Substance Use (ASI) at 3 months in three studies. (2) Meta‐analysis forest plots comparing the time effects of Seeking Safety on Substance Use (ASI) at 3 months in three studies**.

Six studies that measured the outcomes using the same scales were included in the first meta‐analysis. Three of these studies used the Addiction Severity Index (ASI) as a substance use measure at 3‐month follow‐up (Boden et al., 2012; Najavits et al., 2018; Schafer et al., 2019). The other three studies used the Clinician Assessed PTSD Scale (CAPS) version 1 (Blake, 1990; Blake et al., 1995) as a PTSD measure at 6‐month follow‐up (Hien et al., 2004, 2009; Zlotnick et al., 2009). Figures 2 and 3 show the forest plots from the meta‐analysis on the time, group, and time by group effects of SS on PTSD symptom severity at 6 months and addiction severity at 3 months, respectively, in the corresponding studies (see also Table 3). The pooled effect SMD (or ES) were group = −0.17, time = −1.06, and time by group = −0.76 (*p* = .002) for CAPS‐1 at 6‐month follow‐up. There was significant heterogeneity found with the time effect. The pooled SMD (or ES) were group = −0.2 (*p* = .04), time = −0.29, and time by group = −0.24 for ASI at 3‐month follow‐up. There was significant heterogeneity found with the time by group effect. Based on the pooled ES, SS has a small effect on CAPS‐1 outcomes by group but a large effect by time and a significant and medium time by group effect. The *Q* and *I* ^2^ statistics rejected heterogeneity for the group and time by group effects but not for the time effects. Based on the pooled SMD (or ES), SS has a medium effect on substance use outcomes by group, by time significantly, and time by group. The *Q* and *I* ^2^ statistics rejected heterogeneity for group or time effects but not for the group by time effects. Together, these findings suggest that SS is effective in reducing PTSD symptoms (measured by CAPS‐1) at 6 months postintervention and substance use (measured by ASI) at 3 months postintervention.

**TABLE 3:** Meta‐analysis results comparing the group, time, and time by group effects of Seeking Safety on substance use (ASI) at 3 months and PTSD symptom severity (CAPS‐1) at 6 months in three studies

Outcomes	Comparisons	*I* ^2^	*τ* ^2^	*p* (heterogeneity)	Standardized mean difference	95% CI	*p* (random effects)
ASI at 3 months	Group	51%	0.0402	.13	−0.20	[−0.88; 0.48]	.34
	Time	0%	0.0030	.73	−0.29	[−0.55; −0.03]	.04^a^
	Group by time	94%	0.7572	<.01^b^	−0.24	[−2.50; 2.01]	.69
CAPS‐1 at 6 months	Group	0%	0.0011	.86	−0.17	[−0.36; 0.02]	.06
	Time	91%	0.3460	<.01^b^	−1.06	[−2.65; 0.53]	.10
	Group by time	0%	0.0005	.87	−0.76	[−0.92; −0.60]	.002^b^

^a^ *p* < .05.

^b^ *p* < .01.

ASI, Addiction Severity Index; CAPS‐1, Clinician Assessed PTSD Scale version 1; PTSD, post‐traumatic stress disorder.

Due to the variety of measures used to assess PTSD and substance use (see Table 2 for details) the second meta‐analysis included all studies that measured PTSD symptoms or substance use regardless of the measures used. Figure S1 shows the forest plots from the meta‐analysis on the time, group, and time by group effects of SS on PTSD symptoms or substance use postintervention, at 3‐month, 6‐month, and 9‐month follow‐up. The pooled effect sizes for time, group, and time by group were summarized in Table S1 and stratified by the intervention version. Table S2 showed the meta‐regression results examining if the intervention version was associated with time, group, and time by group effects on PTSD symptoms or substance use postintervention, at 3‐month, 6‐month, and 9‐month follow‐up. Based on the pooled ES, SS had small to medium effects on substance use by time, group, or time by group. Based on the pooled ES, SS had medium to large effects on PTSD symptoms by time, group, or time by group (except for the group effect at 3‐month follow‐up). The effects on substance use were significant by time at 3 months (*p* = .008) and 6 months (*p* = .02) but not for other time points, by group, or for time by group interactions (see Table S1). As for PTSD, significant effects were found at immediately postintervention, at 6 months, and 9 months by group (*p* = .003, *p* = .0005, *p* = .002, respectively), time (*p* = .001, *p* = .0001, *p* = .002, respectively) and time by group interactions (*p* = .02, *p* = .02, *p* = .0004, respectively). However, for PTSD, significant effects were found at 3 months only by time (*p* = .003)—no significant effects were found by group (*p* = .08) or time by group (*p* = .37) for PTSD at 3 months (see Table S1). Significant heterogeneities were usually found for time or group by time effects and sometimes varied by the intervention version. Meta‐regressions showed that using the full version of SS was significantly associated with decreased effect sizes (negative) and thus better group by time effects in decreasing PTSD symptoms immediately postintervention, and group effects in decreasing substance use at 3‐month follow‐up (see Supplemental Material for details). However, at all other time points, no statistically significant differences were found in the effects between the full and abbreviated versions.

## 4 DISCUSSION

A wealth of literature detailing the effect of SS on PTSD and substance use has been published since the most recent meta‐analyses that reviewed RCTs of SS prior to 2015 (Safety, 2020). Those reviews reported analyses either stratified by group or individual delivery format and did not report findings related to substance use separately from alcohol use (Roberts et al., 2016) or did not examine differences over time (Lenz et al., 2016). Additionally, neither meta‐analysis examined dose‐response of SS to identify if desired health outcomes, for example, decreased substance use or PTSD symptoms, could be attained with abbreviated versions of SS (Lenz et al., 2016; Roberts et al., 2016). Our meta‐analysis revealed seven RCTs that reported PTSD symptoms and substance use outcomes—of which four employed an abbreviated version of SS, administering 12 to 16 of the 25 available topics, as selected by a clinician. Sessions lasted from 1 to 1.5 h depending on the study—making specific intervention dose, including session topics and duration, unclear. Additionally, SS has been delivered using many different modalities, including group and individual sessions, delivered by clinicians and laypeople, and in some studies, combined with other intervention, such as treatment as usual and weekly individual case management. This variance in delivery raises interesting questions about (a) how many sessions are needed, (b) which SS topics are most impactful, and (c) what the minimum length of a session can be to reach the desired outcomes. Such challenges are typical for an intervention such as SS, which has been designed for flexibility across many different implementation contexts. Thus, in this meta‐analysis, we sought to examine the effect of SS on PTSD symptoms and substance use across studies (time, group, and time by group comparisons), and examine the dose‐response of SS by comparing the effects of the full version to a collection of abbreviated versions of SS across studies.

Our findings confirmed substantial variation in the delivery and dose across studies and topical content of SS. Our meta‐analysis findings indicate that SS effectively reduced PTSD symptom severity (via CAPS‐1) at 6 months postintervention and substance use (via ASI) at 3 months postintervention. When examining the effect of SS on the reduction of PTSD symptoms and substance use across multiple different measures, SS had medium to large effects on PTSD by time, group, or time by group and small to medium effects on substance use by time, group, or time by group (except for the group effect at 3‐month follow‐up). These findings diverge from the most recent meta‐analysis conducted in 2016 (Roberts et al., 2016). Roberts et al. (2016) found no significant improvements in PTSD symptom severity or reduction in substance use when comparing SS against “usual care/minimal intervention or against another active psychological therapy” (p. 35), with the exception of a moderate reduction in substance use for full dose SS immediately postintervention. This difference in findings may be associated with the method of analysis and grouping conducted in the Roberts et al. (2016) study. While they conducted a post hoc analysis including only studies that examined the full version of SS, we included studies examining the effect of abbreviated dose and full dose versions of SS. We then expanded our understanding of the varying effect of abbreviated versus full dose versions of SS through meta‐regression. Meta‐regression findings suggest that abbreviated dose versions of SS are generally functioning as well as the full dose version of SS when observing long‐term effects (greater than 3 months).

Overall, our findings indicate that SS may be effective at reducing PTSD symptoms and substance use; however, SS may be more effective in reducing PTSD symptoms than substance use. This finding is convergent with the literature on PTSD/SUD treatment studies, which consistently find that it is easier to obtain reduction in PTSD than SUD (Najavits & Hien, 2013; Najavits et al., 2020). This has been found across the board, regardless of model (e.g., see RCTs by Back et al., 2019; Coffey et al., 2016; McGovern et al., 2011; Mills et al., 2012). Moreover, SUD‐only models have been found to reduce PTSD as much as PTSD or PTSD/SUD models in various RCTs (e.g., Foa et al., 2013; Hien et al., 2004; McGovern et al., 2015; Sannibale et al., 2013; Schafer et al., 2019). There are various reasons that PTSD is easier to treat than SUD. SUD is widely understood as a chronic relapsing disorder that represents a lifelong commitment to sobriety whereas PTSD is conceptualized as a disorder that is more amenable to short‐term treatment (McLellan et al., 2005). Second, SUD is often characterized as a “disease of denial” in that it is highly likely to be minimized and disavowed, often causing suffering to those adjacent to the person, but requiring significant consequences and “hitting bottom” before it is recognized by the individuals themselves. In contrast, PTSD typically causes very direct and noticeable suffering of which the individual is aware. Notably, patients with both PTSD and SUD want treatment of PTSD more than they want treatment of SUD (Najavits et al., 2004), which has also been replicated among patients with comorbid PTSD and gambling disorder (Najavits, 2003).

Another key finding is that the long‐term effects of abbreviated versions of SS are similar to the full dose version of SS. Thus, abbreviated SS may represent a strong choice as it takes less time for both patients and providers. More research is needed to identify if pairing SS with additional PTSD and/or substance use treatments, such as pharmacological treatments (Hoskins et al., 2021; Norman et al., 2012), trauma‐focused treatments like cognitive processing therapy (CPT) (Asmundson et al., 2019), or complementary and integrative health (CIH) treatments, such as trauma‐informed yoga (Kelly et al., 2021; Murphy et al., 2019), can improve patient outcomes.

Lastly, we note that there were inconsistencies in the included studies’ measures and timepoints for assessing PTSD and substance use, which rendered a more detailed dose‐response curve analysis unfeasible. Future research would benefit from use of common data elements (e.g., standardized measures, data assessment timepoints) to improve interpretability and comparability of research findings, and calculation of detailed dose‐response across studies. Leading organizations, including the National Institutes of Health, emphasize the need for greater protocol publishing, standards of research (establishing common data elements), and open data repositories for research studies to improve process sharing, eliminate redundancies, and improve reproducibility, replicability, and generalizability of research (National Academies of Sciences, 2019).

### 4.1 Strengths and limitations

This meta‐analysis is the first to evaluate the dose effect of SS for treatment of PTSD and SUD using RCTs. All the RCTs included in this evaluation of the effects of SS had low risk of bias, which adds considerable strength to the evidence of the effectiveness of SS in the treatment of comorbid PTSD and SUD. However, there are several limitations. In one case (Hien et. al, 2009), the data we used were the raw data provided by the author that allowed us to rerun the descriptive statistics (i.e., sample sizes, means and standard deviations of CAPS at 3 months postintervention). Therefore, there is potential replication bias. We found that the replicated baseline descriptive statistics were the same as the published numbers while the sample sizes at 3 months postintervention were fewer than the reported sample sizes in the published consort table for the control and intervention groups, as the intention‐to‐treat method was not replicated. While heterogeneity was not evident for the group and time by group effects, there was significant heterogeneity in the time effects. The heterogeneity could come from the designs of intervention versus control, sample characteristics, and measures. The implementation of SS among the studies did vary (e.g., abbreviated SS, SS with treatment as usual); so too there was no single comparison used in all study designs (e.g., treatment as usual, women's health education). It is possible that this inconsistency could obfuscate treatment results. Differences in the effectiveness of SS based on sociodemographic factors, particularly those representative of multiply marginalized identities (e.g., race, gender, sexual orientation), were not evaluated. However, we do note that SS has been particularly notable in being studied in real‐world community‐based settings, with frontline providers, and a high level of minority representation. Moreover, in the meta‐analysis, the time and time by group SMD (or ES) were calculated based on the mean and standard deviations of the intervention and control groups at baseline or follow‐ups as independent groups while the pre‐/postcorrelations were unknown and thus not considered in the computation.

## 5 CONCLUSION

Our findings suggest that SS is an effective intervention for the comorbid treatment PTSD and SUD across various settings and among diverse populations. Importantly, the long‐term effects of abbreviated versions of SS are comparable to those of the full version of SS. Our findings also replicate the consistent finding in the literature that it is easier to see improvement in PTSD than SUD; this has been found across models studied thus far for comorbid PTSD and SUD. Finally, we note that inconsistencies across studies in design, length of intervention, and number of SS sessions delivered means that it is not possible to identify a minimum effective dose for SS. We suggest that future studies of PTSD/SUD should use standardized measurement tools and data assessment time points, and report dose responses for their studies.

## FUNDING

Research reported in this publication was supported by the National Institute of Nursing Research of the National Institutes of Health under Award Number K23NR020208 (A. D. F. Sherman). The content is solely the responsibility of the authors and does not necessarily represent the official views of the National Institutes of Health.

## CONFLICT OF INTEREST STATEMENT

No competing financial interests exist.

### PEER REVIEW

The peer review history for this article is available at https://publons.com/publon/10.1002/brb3.2999.

## Supporting information

Supplementary information

## Data Availability

No primary data were created as part of this review. All data analyzed come from published manuscripts.
