# Supplementary material for: Seeking safety intervention for comorbid post‐traumatic stress and substance use disorder: A meta‐analysis
Source: Brain Behav. 2023 Apr 10;13(5):e2999. doi: 10.1002/brb3.2999 (PMC10175993; doi:10.1002/brb3.2999)
Supplement: Supplementary file 1 — Supplementary information [file BRB3-13-e2999-s001.docx]

Supplemental Table 1. Meta-analysis results comparing the group, time, and time by group effects of Seeking Safety on substance use and PTSD measures from baseline to post intervention, at 3-, 6-, and 9-month follow-up and by Seeking Safety version (full versus abbreviated)

| Outcomes | Comparisons | I^2^ | | | τ^2^ | | | P (heterogeneity) | | | Standardized mean difference | | | 95% CI | | | k | | | P (random effect) |
| --- | --- | --- | --- | --- | --- | --- | --- | --- | --- | --- | --- | --- | --- | --- | --- | --- | --- | --- | --- | --- |
|  | Version | Full | Condensed | All | Full | Condensed | All | Full | Condensed | All | Full | Condensed | All | Full | Condensed | All | Full | Condensed | All | All |
| Substance use post intervention | Group | - | 0% | 70% | - | 0.0065 | 0.1345 | - | 0.44 | 0.04* | -0.71 | -0.03 | -0.21 | [-1.19; -0.23] | [-1.29; 1.22] | [-1.28; 0.85] | 1 | 2 | 3 | 0.48 |
|  | Time | - | 0% | 0% | - | 0.0020 | 0.0008 | - | 0.57 | 0.85 | -0.10 | -0.14 | -0.13 | [-0.54; 0.33] | [-1.02; 0.75] | [-0.32; 0.06] | 1 | 2 | 3 | 0.10 |
|  | Group by time | - | 97% | 96% | - | 2.1826 | 1.9564 | - | ** | ** | -1.26 | 0.39 | -0.16 | [-1.77; 0.75] | [-13.22; 14.00] | [-3.72; 3.40] | 1 | 2 | 3 | 0.86 |
| PTSD post intervention | Group | 0% | 18% | 0% | 0.0062 | 0.0311 | 0.0203 | 0.79 | 0.30 | 0.63 | -0.38 | -0.20 | -0.30 | [-0.58; -0.19] | [-0.74; 0.34] | [-0.46; -0.14] | 5 | 3 | 8 | 0.003** |
|  | Time | 29% | 0% | 33% | 0.0385 | 0.0038 | 0.0357 | 0.23 | 0.58 | 0.17 | -0.58 | -0.32 | -0.46 | [-0.91; -0.24] | [-0.60; -0.03] | -0.67; -0.25] | 5 | 3 | 8 | 0.001** |
|  | Group by time | 80% | 93% | 93% | 0.2867 | 0.2866 | 0.6194 | ** | ** | ** | -1.35 | -0.18 | -0.90 | [-2.09; -0.61] | [-1.61; 1.25] | [-1.59; -0.21] | 5 | 3 | 8 | 0.02* |
| Substance use at 3 months | Group | - | 0% | 30% | - | 0.0009 | 0.0379 | - | 0.84 | 0.23 | -0.52 | -0.04 | -0.14 | [-0.96; -0.08] | [-0.21; 0.12] | [-0.50; 0.21] | 1 | 3 | 4 | 0.28 |
|  | Time | - | 0% | 0% | - | 0.0002 | 0.0019 | - | 0.92 | 0.86 | -0.42 | -0.24 | -0.26 | [-0.83; 0.00] | [-0.35; -0.13] | [-0.40; -0.13] | 1 | 3 | 4 | 0.008** |
|  | Group by time | - | 93% | 90% | - | 0.3537 | 0.2534 | - | ** | ** | -0.45 | -0.11 | -0.20 | [-0.85; -0.05] | [-1.68; 1.46] | [-1.06; 0.66] | 1 | 3 | 4 | 0.52 |
| PTSD at 3 months | Group | 0% | 0% | 0% | 0.0057 | 0.0022 | 0.0107 | 0.65 | 0.79 | 0.72 | 0.08 | -0.15 | -0.11 | [-0.34; 0.50] | [-0.27; -0.03] | [-0.23; 0.01] | 3 | 5 | 8 | 0.08 |
|  | Time | 11% | 93% | 89% | 0.0287 | 0.2461 | 0.1635 | 0.32 | ** | ** | -0.53 | -0.80 | -0.72 | [-1.19; 0.13] | [-1.45; -0.14] | [-1.09; -0.34] | 3 | 5 | 8 | 0.003** |
|  | Group by time | 0% | 97% | 96% | 0.0012 | 0.8816 | 0.4949 | 0.85 | ** | ** | -0.18 | -0.30 | -0.25 | [-0.42; 0.07] | [-1.49; 0.89] | [-0.86; 0.37] | 3 | 5 | 8 | 0.37 |
| Substance use at 6 months | Group | 46% | 0% | 16% | 0.0820 | <0.0001 | 0.0367 | 0.16 | 0.79 | 0.31 | -0.30 | -0.16 | -0.23 | [-1.22; 0.61] | [-0.49; 0.16] | [-0.53; 0.06] | 3 | 2 | 5 | 0.09 |
|  | Time | 33% | 0% | 0% | 0.0385 | 0.0034 | 0.0184 | 0.23 | 0.39 | 0.42 | -0.37 | -0.29 | -0.32 | [-1.08; 0.34] | [-1.29; 0.70] | [-0.55; -0.10] | 3 | 2 | 5 | 0.02* |
|  | Group by time | 96% | 0% | 96% | 1.9815 | <0.0001 | 1.6354 | ** | 0.96 | ** | 0.10 | -1.40 | -0.51 | [-3.49; 3.69] | [-1.46; -1.34] | [-2.13; 1.11] | 3 | 2 | 5 | 0.43 |
| PTSD at 6 months | Group | 0% | 0% | 0% | 0.0200 | 0.0024 | 0.0119 | 0.58 | 0.69 | 0.78 | -0.24 | -0.22 | -0.23 | [-0.43; -0.05] | [-0.37; -0.07] | [-0.33; -0.13] | 8 | 4 | 12 | 0.0005*** |
|  | Time | 0% | 95% | 85% | 0.0188 | 0.3741 | 0.1381 | 0.58 | ** | ** | -0.55 | -0.89 | -0.69 | [-0.73; -0.37] | [-1.89; 0.11] | [-0.96; -0.42] | 8 | 4 | 12 | 0.0001*** |
|  | Group by time | 95% | 98% | 96% | 1.2502 | 1.0874 | 1.1230 | ** | ** | ** | -0.97 | -0.60 | -0.84 | [-1.94; -0.01] | [-2.27; 1.08] | [-.53; -0.15] | 8 | 4 | 12 | 0.02* |
| Substance use at 9 months | Group | 0% | - | 0% | 0.0092 | - | 0.0092 | 0.45 | - | 0.45 | -0.33 | - | -0.33 | [-2.13; 1.47] | - | [-2.13; 1.47] | 2 | 0 | 2 | 0.26 |
|  | Time | 57% | - | 57% | 0.0818 | - | 0.0818 | 0.13 | - | 0.13 | -0.24 | - | -0.24 | [-3.73; 3.24] | - | [-3.73; 3.24] | 2 | 0 | 2 | 0.54 |
|  | Group by time | 67% | - | 67% | 0.1274 | - | 0.1274 | 0.08 | - | 0.08 | 0.19 | - | 0.19 | [-3.94; 4.32] |  | [-3.94; 4.32] | 2 | 0 | 2 | 0.66 |
| PTSD at 9 months | Group | 0% | - | 0% | 0.0231 | - | 0.0231 | 0.59 | - | 0.59 | -0.45 | - | -0.45 | [-0.66; -0.24] | - | [-0.66; -0.24] | 7 | 0 | 7 | 0.002** |
|  | Time | 70% | - | 70% | 0.1410 | - | 0.1410 | ** | - | ** | -0.85 | - | -0.85 | [-1.26; -0.44] | - | [-1.26; -0.44] | 7 | 0 | 7 | 0.002** |
|  | Group by time | 79% | - | 79% | 0.3589 | - | 0.3589 | ** | - | ** | -1.75 | - | -1.75 | [-2.37; -1.13] | - | [-2.37; -1.13] | 7 | 0 | 7 | 0.0004*** |

*p<0.05, **p<0.01, ***p<.001.

Supplemental Table 2. Meta-regression results predicting the group, time, and time by group effects of Seeking Safety on substance use and PTSD measures from baseline to post intervention, at 3-, 6- and 9-month follow-ups by seeking safety version (full versus abbreviated)

| Outcomes | Comparisons | Variables | Estimate | SE | t | df | p | 95% CI |
| --- | --- | --- | --- | --- | --- | --- | --- | --- |
| Substance use post intervention | Group | Intercept | -0.0198 | 0.1069 | -0.1848 | 1 | 0.8837 | [-1.3784; 1.3389] |
|  |  | Version  (Full=1, Condensed=0) | -0.6912 | 0.2113 | -3.2714 | 1 | 0.1889 | [-3.3761; 1.9936] |
|  | Time | Intercept | -0.1386 | 0.0692 | -2.0031 | 1 | 0.2948 | [-1.0179; 0.7406] |
|  |  | Version  (Full=1, Condensed=0) | 0.0344 | 0.1421 | 0.2418 | 1 | 0.8489 | [-1.7712; 1.8399] |
|  | Group by time | Intercept | 0.3872 | 1.0710 | 0.3615 | 1 | 0.7792 | [-13.2217; 13.9961] |
|  |  | Version  (Full=1, Condensed=0) | -1.6499 | 1.8575 | -0.8882 | 1 | 0.5376 | [-25.2518; 21.9520] |
| PTSD post intervention | Group | Intercept | -0.2092 | 0.0885 | -2.3637 | 6 | 0.0560 | [-0.4258; 0.0074] |
|  |  | Version  (Full=1, Condensed=0) | -0.1736 | 0.1253 | -1.3859 | 6 | 0.2151 | [-0.4801; 0.1329] |
|  | Time | Intercept | -0.3120 | 0.1139 | -2.7394 | 6 | 0.0338* | [-0.5908; -0.0333] |
|  |  | Version  (Full=1, Condensed=0) | -0.2665 | 0.1543 | -1.7272 | 6 | 0.1349 | [-0.6440; 0.1110] |
|  | Group by time | Intercept | -0.1765 | 0.3301 | -0.5347 | 6 | 0.6121 | [-0.9841; 0.6311] |
|  |  | Version  (Full=1, Condensed=0) | -1.1753 | 0.4252 | -2.7638 | 6 | 0.0327* | [-2.2158; -0.1348] |
| Substance use at 3 months | Group | Intercept | -0.0442 | 0.0392 | -1.1270 | 2 | 0.3768 | [-0.2129; 0.1245] |
|  |  | Version  (Full=1, Condensed=0) | -0.4752 | 0.1003 | -4.7359 | 2 | 0.0418* | [-0.9069; -0.0435] |
|  | Time | Intercept | -0.2385 | 0.0251 | -9.5154 | 2 | 0.0109* | [-0.3464; -0.1307] |
|  |  | Version  (Full=1, Condensed=0) | -0.1776 | 0.0662 | -2.6839 | 2 | 0.1153 | [-0.4624; 0.1071] |
|  | Group by time | Intercept | -0.1075 | 0.3650 | -0.2945 | 2 | 0.7961 | [-1.6777; 1.4628] |
|  |  | Version  (Full=1, Condensed=0) | -0.3411 | 0.7354 | -0.4637 | 2 | 0.6884 | [-3.5054; 2.8233] |
| PTSD at 3 months | Group | Intercept | -0.1545 | 0.0444 | -3.4788 | 6 | 0.0132* | [-0.2632; -0.0458] |
|  |  | Version  (Full=1, Condensed=0) | 0.2312 | 0.1023 | 2.2596 | 6 | 0.0646 | [-0.0192; 0.4815] |
|  | Time | Intercept | -0.7991 | 0.2033 | -3.9310 | 6 | 0.0077 | [-1.2965; -0.3017] |
|  |  | Version  (Full=1, Condensed=0) | 0.2454 | 0.3507 | 0.6998 | 6 | 0.5103 | [-0.6127; 1.1036] |
|  | Group by time | Intercept | -0.2881 | 0.3503 | -0.8225 | 6 | 0.4423 | [-1.1451; 0.5690] |
|  |  | Version  (Full=1, Condensed=0) | 0.1061 | 0.5831 | 0.1819 | 6 | 0.8616 | [-1.3207; 1.5329] |
| Substance use at 6 months | Group | Intercept | -0.1639 | 0.1580 | -1.0370 | 3 | 0.3760 | [-0.6668; 0.3390] |
|  |  | Version  (Full=1, Condensed=0) | -0.1500 | 0.2335 | -0.6424 | 3 | 0.5663 | [-0.8933; 0.5932] |
|  | Time | Intercept | -0.2973 | 0.1254 | -2.3699 | 3 | 0.0985 | [-0.6964; 0.1019] |
|  |  | Version  (Full=1, Condensed=0) | -0.0684 | 0.1918 | -0.3564 | 3 | 0.7451 | [-0.6788; 0.5421] |
|  | Group by time | Intercept | -1.3963 | 0.8167 | -1.7096 | 3 | 0.1859 | [-3.9955; 1.2029] |
|  |  | Version  (Full=1, Condensed=0) | 1.4861 | 1.0629 | 1.3982 | 3 | 0.2565 | [-1.8964; 4.8687] |
| PTSD at 6 months | Group | Intercept | -0.2164 | 0.0654 | -3.3067 | 10 | 0.0079** | [-0.3622; -0.0706] |
|  |  | Version  (Full=1, Condensed=0) | -0.0230 | 0.0973 | -0.2365 | 10 | 0.8179 | [-0.2399; 0.1938] |
|  | Time | Intercept | -0.8894 | 0.1897 | -4.6888 | 10 | 0.0009*** | [-1.3121; -0.4668] |
|  |  | Version  (Full=1, Condensed=0) | 0.3254 | 0.2425 | 1.3418 | 10 | 0.2093 | [-0.2150; 0.8658] |
|  | Group by time | Intercept | -0.5960 | 0.5527 | -1.0782 | 10 | 0.3063 | [-1.8275; 0.6356] |
|  |  | Version  (Full=1, Condensed=0) | -0.3744 | 0.6825 | -0.5485 | 10 | 0.5954 | [-1.8952; 1.1464] |

*p<0.05, **p<0.01, ***p<0.001.

Supplemental Figure 1. Meta-analysis results comparing the group, time, and time by group effects of Seeking Safety on substance use and PTSD measures from baseline to post intervention, at 3-, 6-, and 9-month follow-ups and by Seeking Safety version (full versus abbreviated)

| Supplemental Figure 1.1. Meta-analysis results comparing the group effects of Seeking Safety on substance use measures from baseline to post intervention  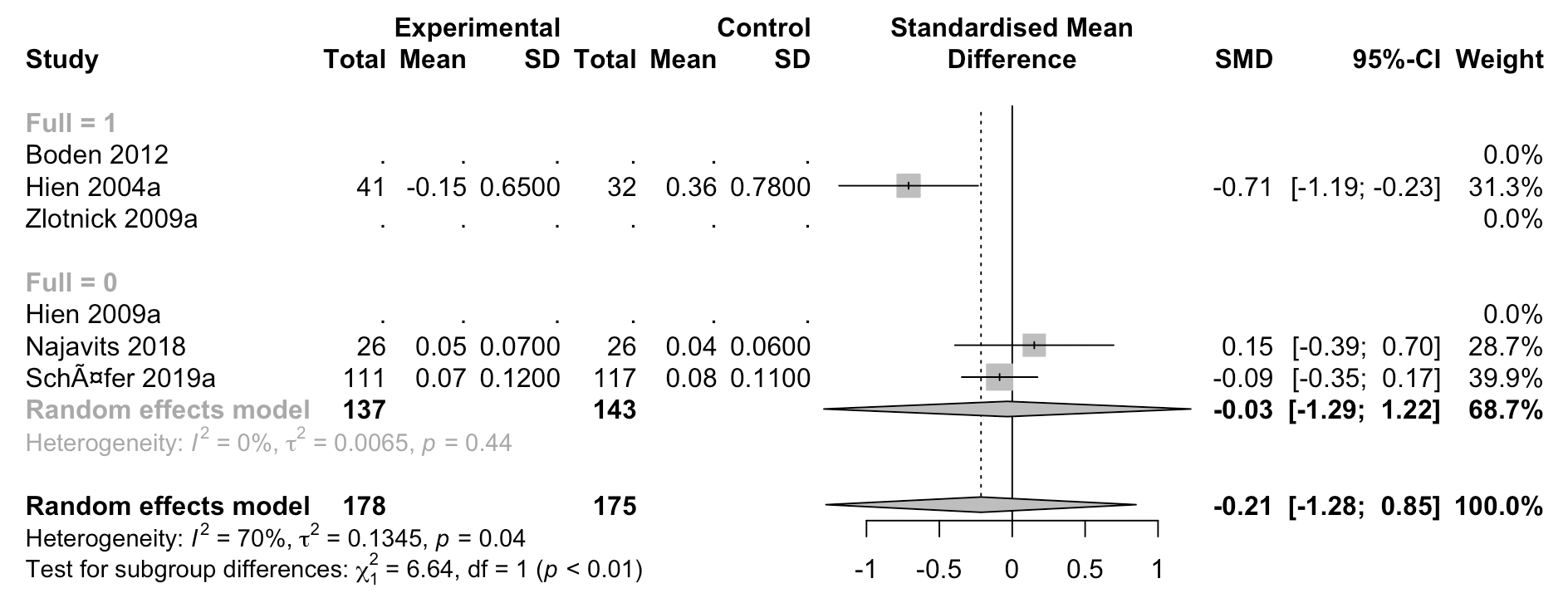 |
| --- |
| Supplemental Figure 1.2. Meta-analysis results comparing the time effects of Seeking Safety on substance use measures from baseline to post intervention  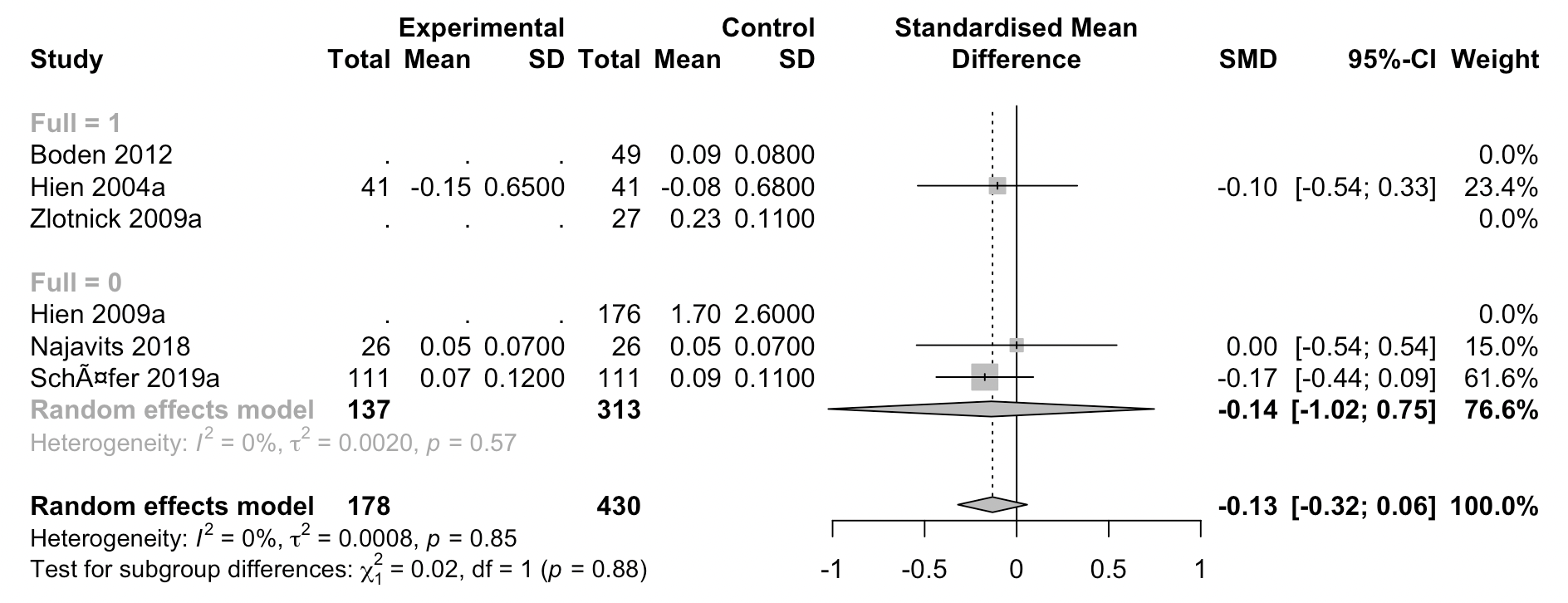 |
| Supplemental Figure 1.3. Meta-analysis results comparing the time by group effects of Seeking Safety on substance use measures from baseline to post intervention  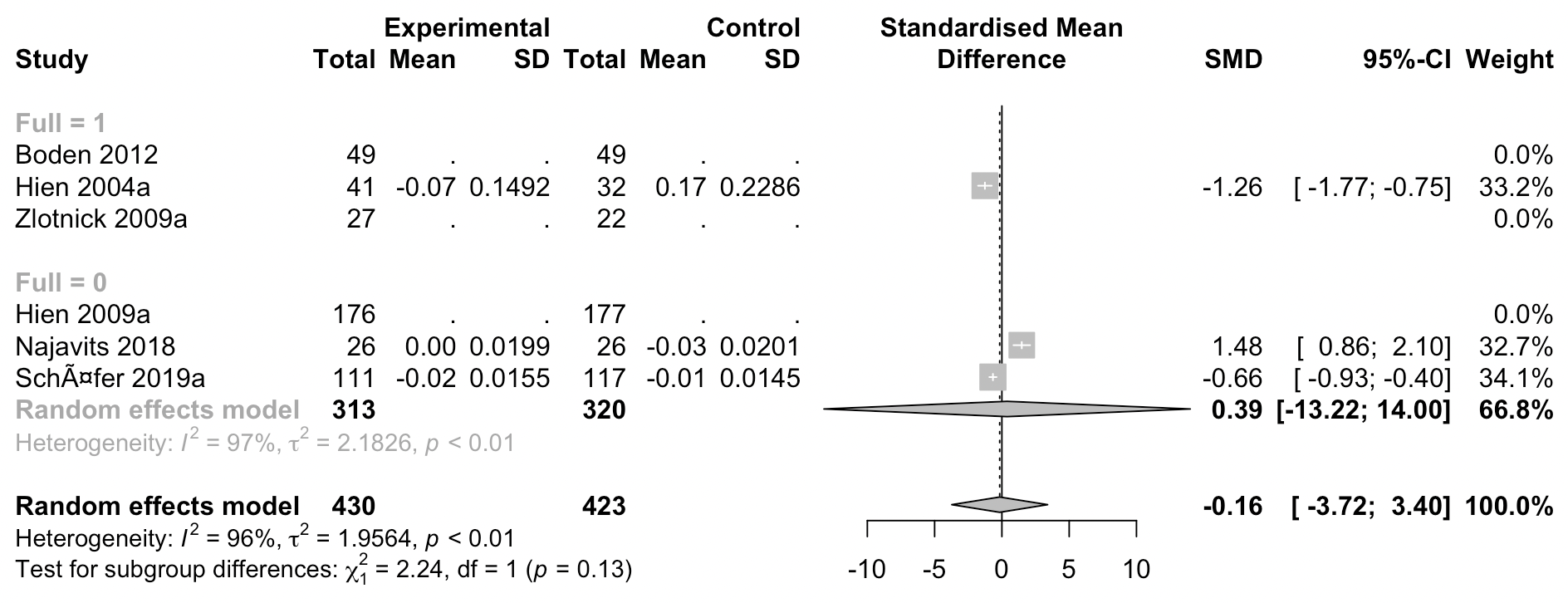 |
| PTSD post intervention |
| Supplemental Figure 1.4. Meta-analysis results comparing the group effects of Seeking Safety on PTSD measures from baseline to post intervention  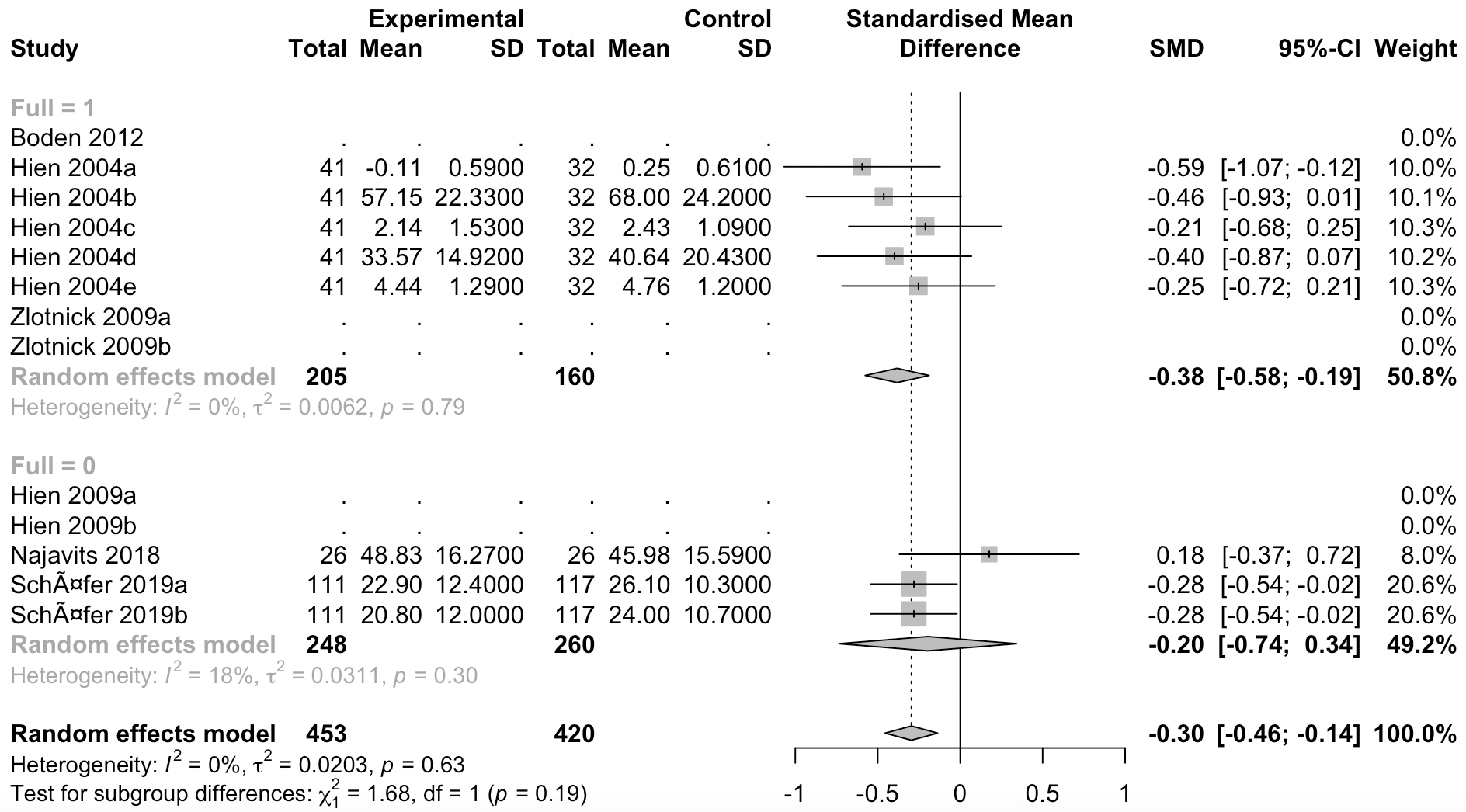 |
| Supplemental Figure 1.5. Meta-analysis results comparing the time effects of Seeking Safety on PTSD measures from baseline to post intervention  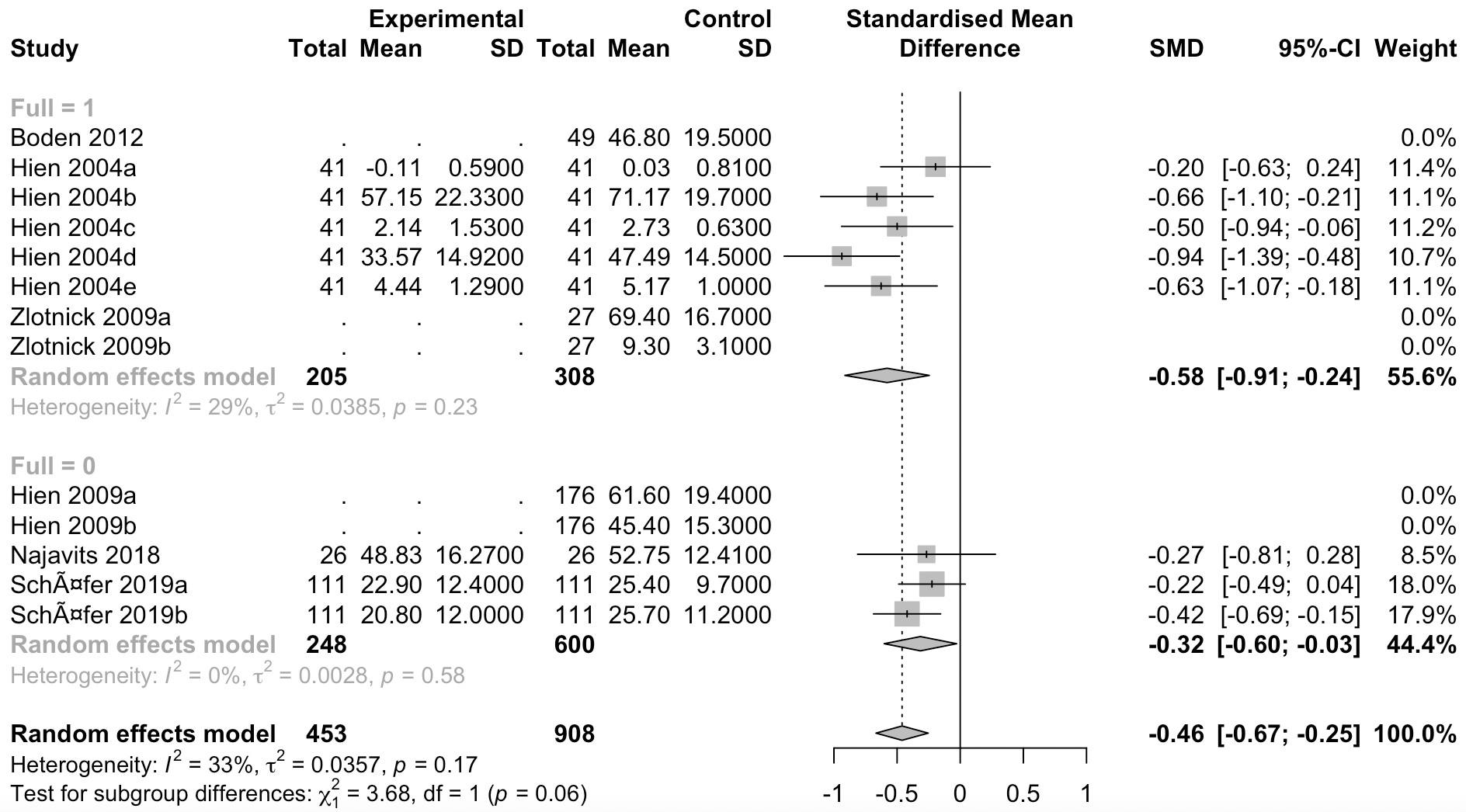 |
| Supplemental Figure 1.6. Meta-analysis results comparing the time by group effects of Seeking Safety on PTSD measures from baseline to post intervention  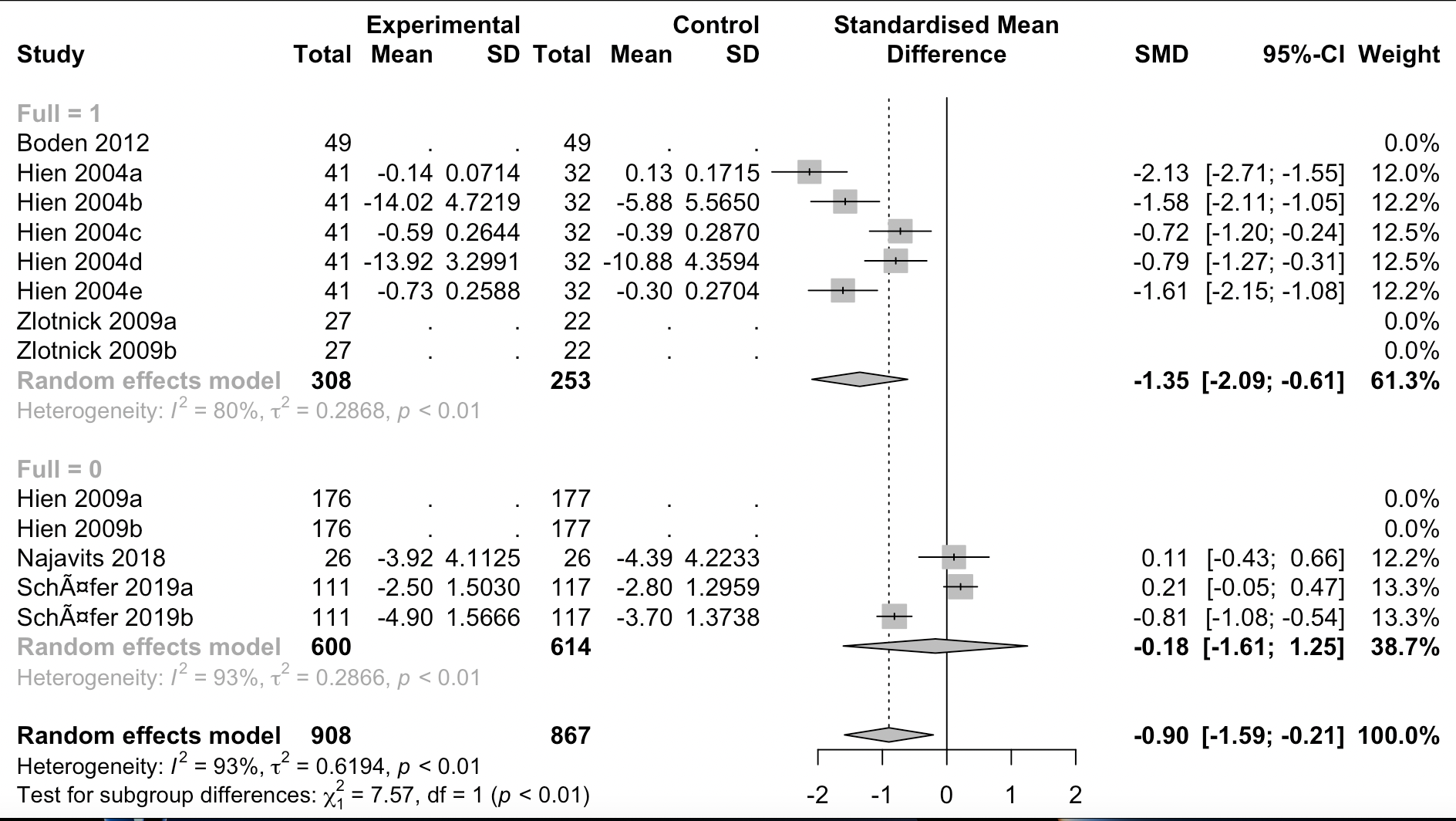 |
| Substance use at 3 months |
| Supplemental Figure 1.7. Meta-analysis results comparing the group effects of Seeking Safety on substance use measures from baseline to 3 months  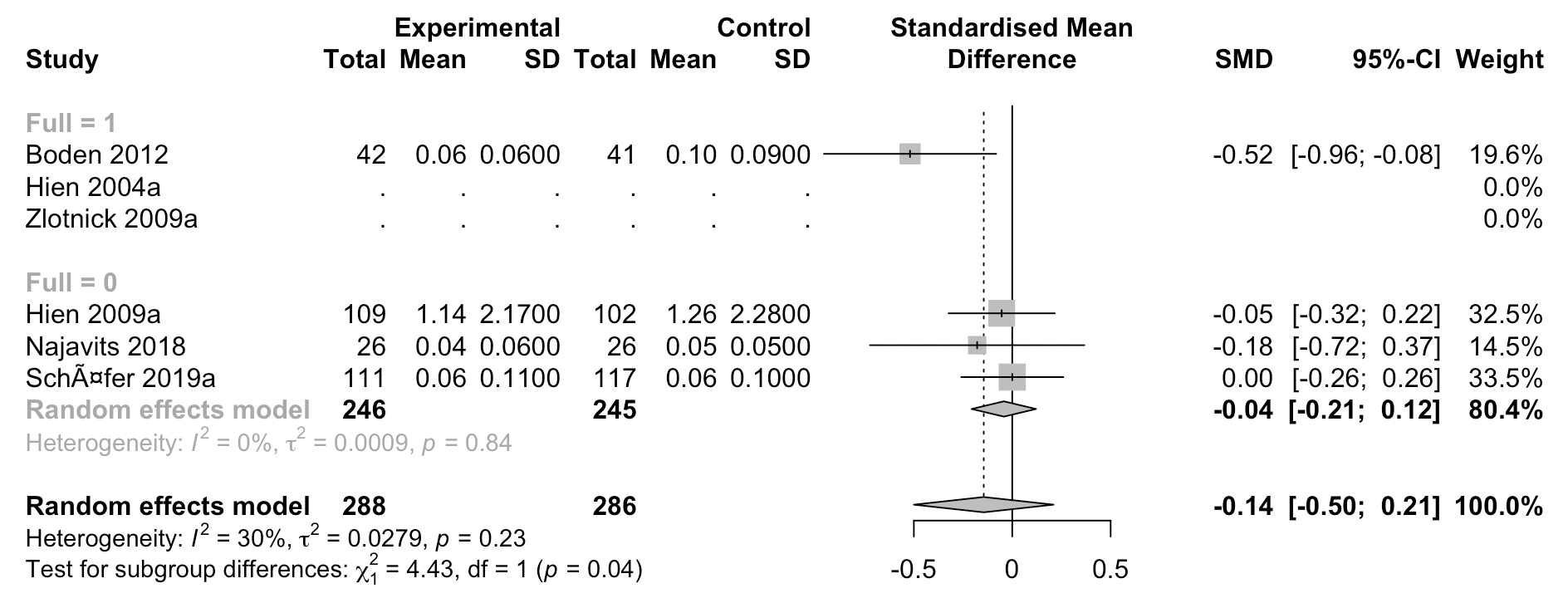 |
| Supplemental Figure 1.8. Meta-analysis results comparing the time effects of Seeking Safety on substance use measures from baseline to 3 months  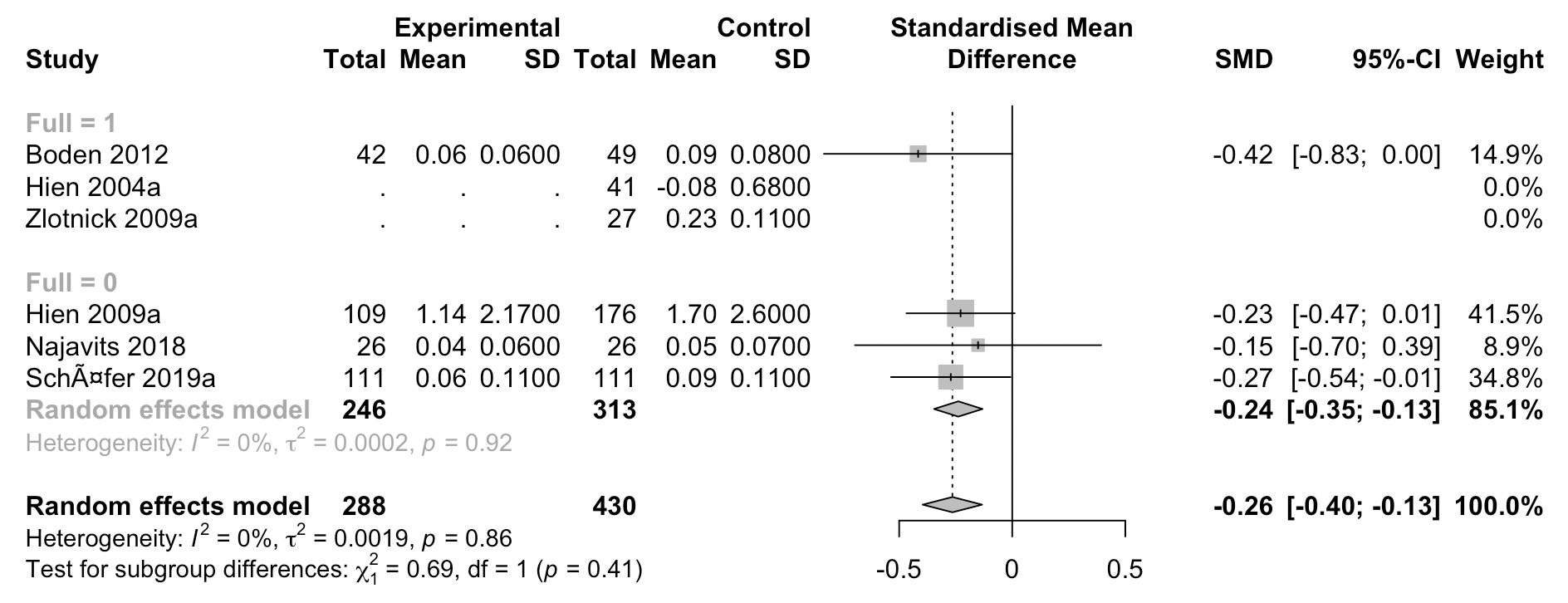 |
| Supplemental Figure 1.9. Meta-analysis results comparing the time by group effects of Seeking Safety on substance use measures from baseline to 3 months  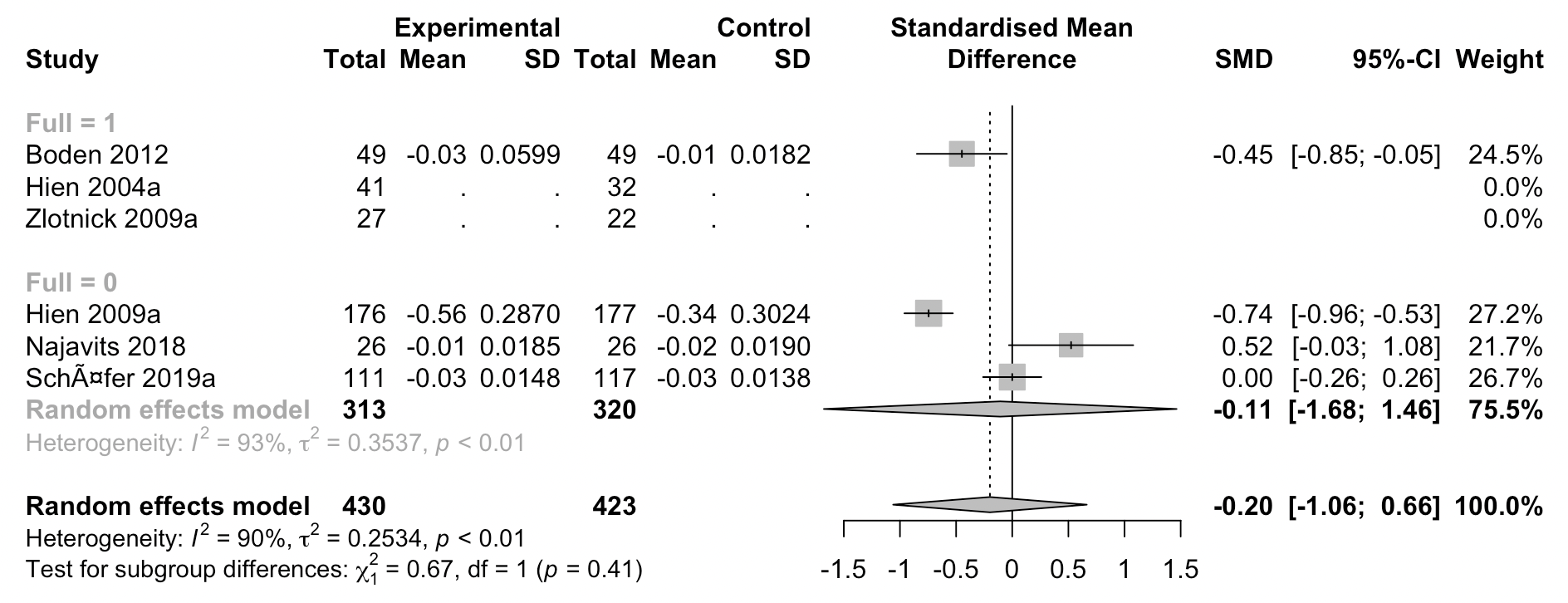 |
| PTSD at 3 months |
| Supplemental Figure 1.10. Meta-analysis results comparing the group effects of Seeking Safety on PTSD measures from baseline to 3 months  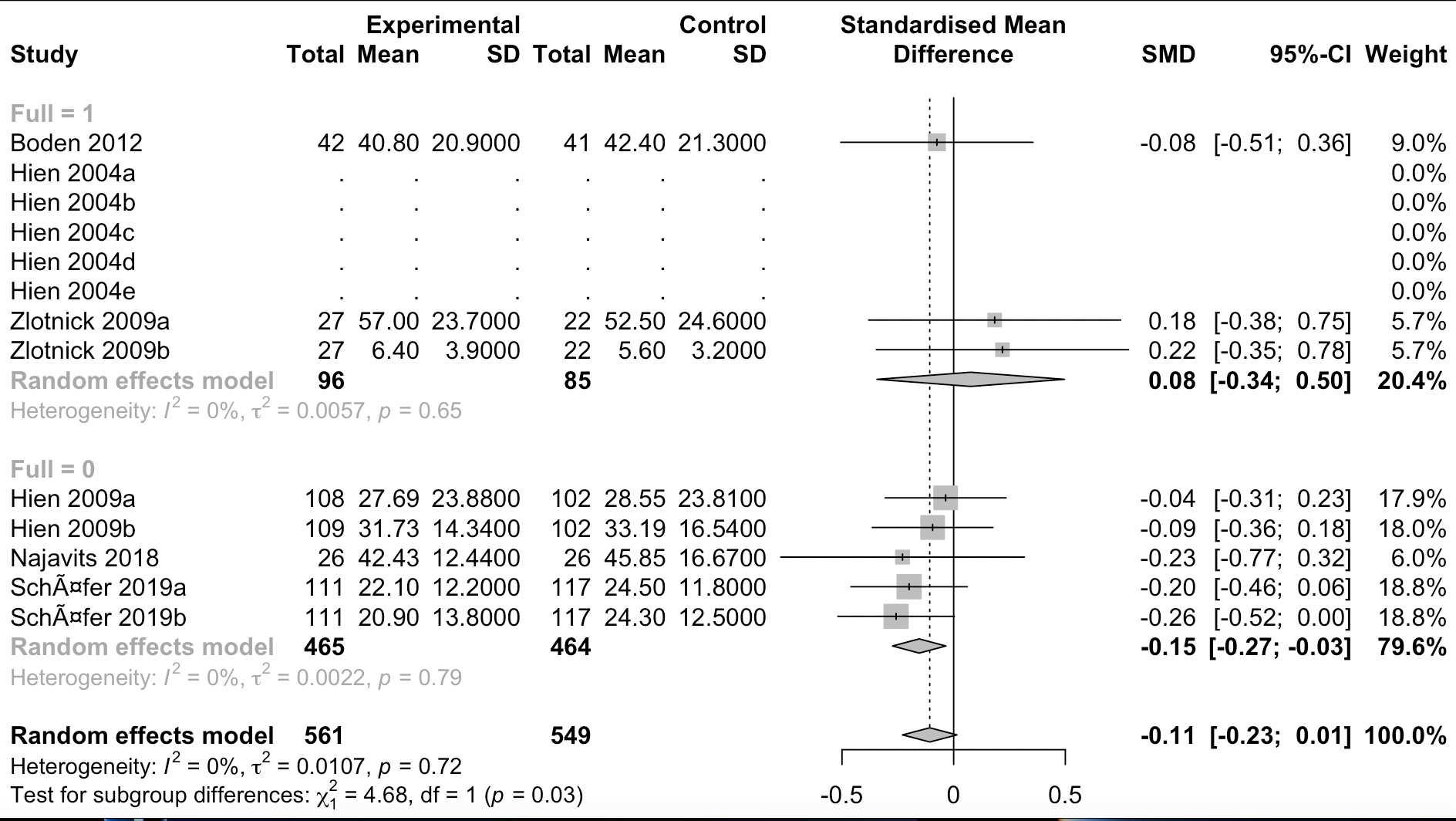 |
| Supplemental Figure 1.11. Meta-analysis results comparing the time effects of Seeking Safety on PTSD measures from baseline to 3 months  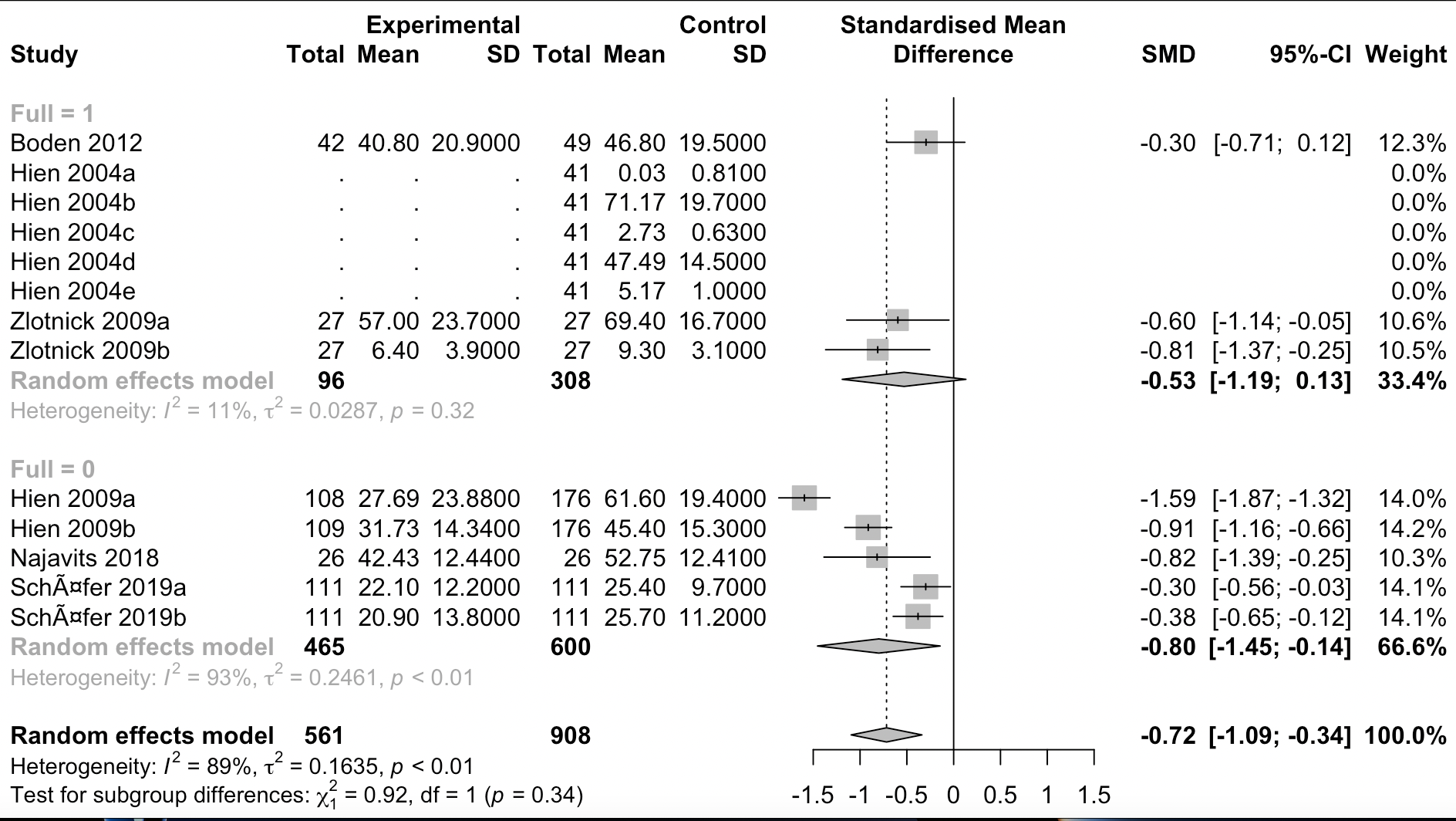 |
| Supplemental Figure 1.12. Meta-analysis results comparing the time by group effects of Seeking Safety on PTSD measures from baseline to 3 months  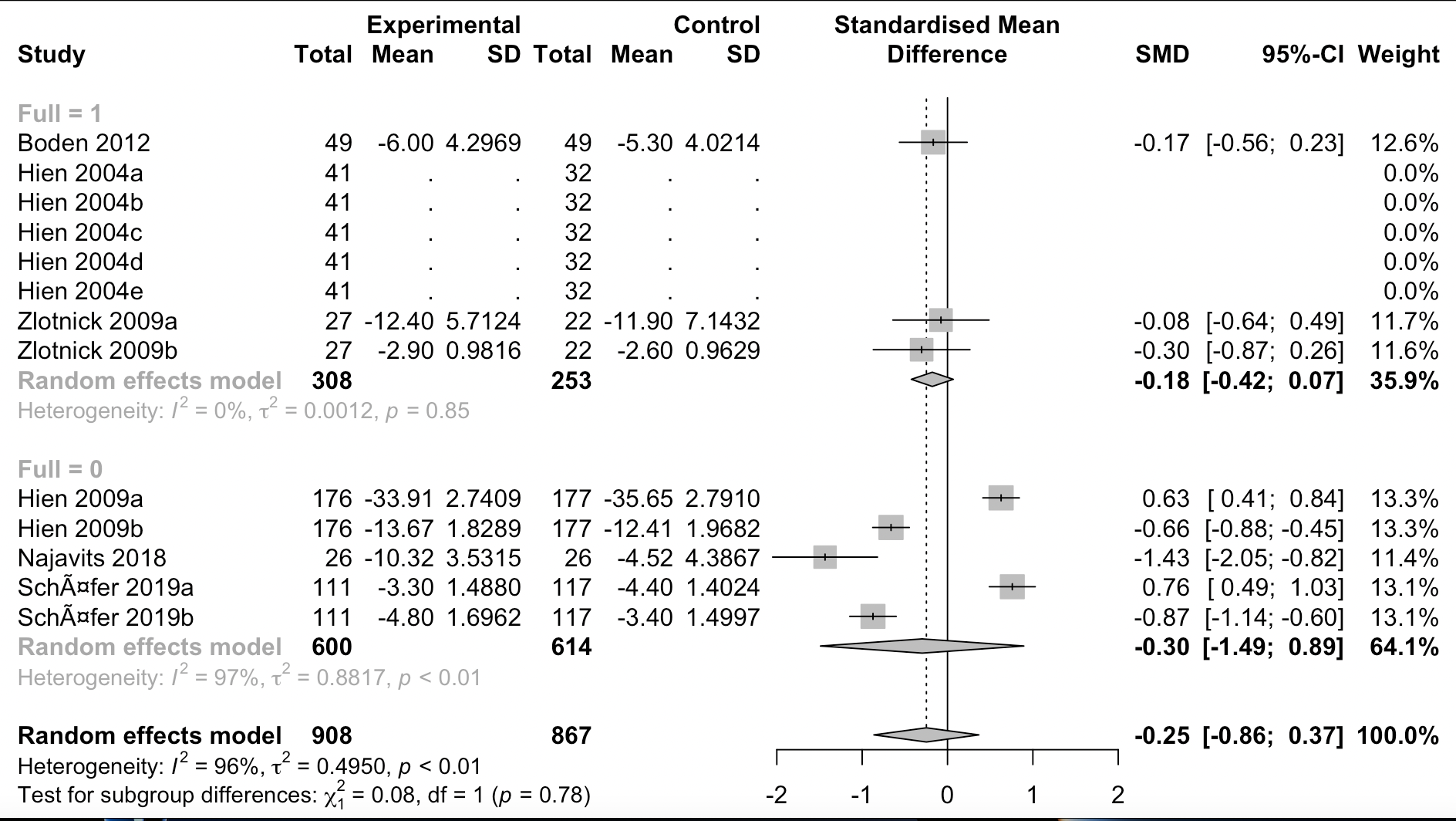 |
| Substance use at 6 months |
| Supplemental Figure 1.13. Meta-analysis results comparing the group effects of Seeking Safety on substance use measures from baseline to 6 months  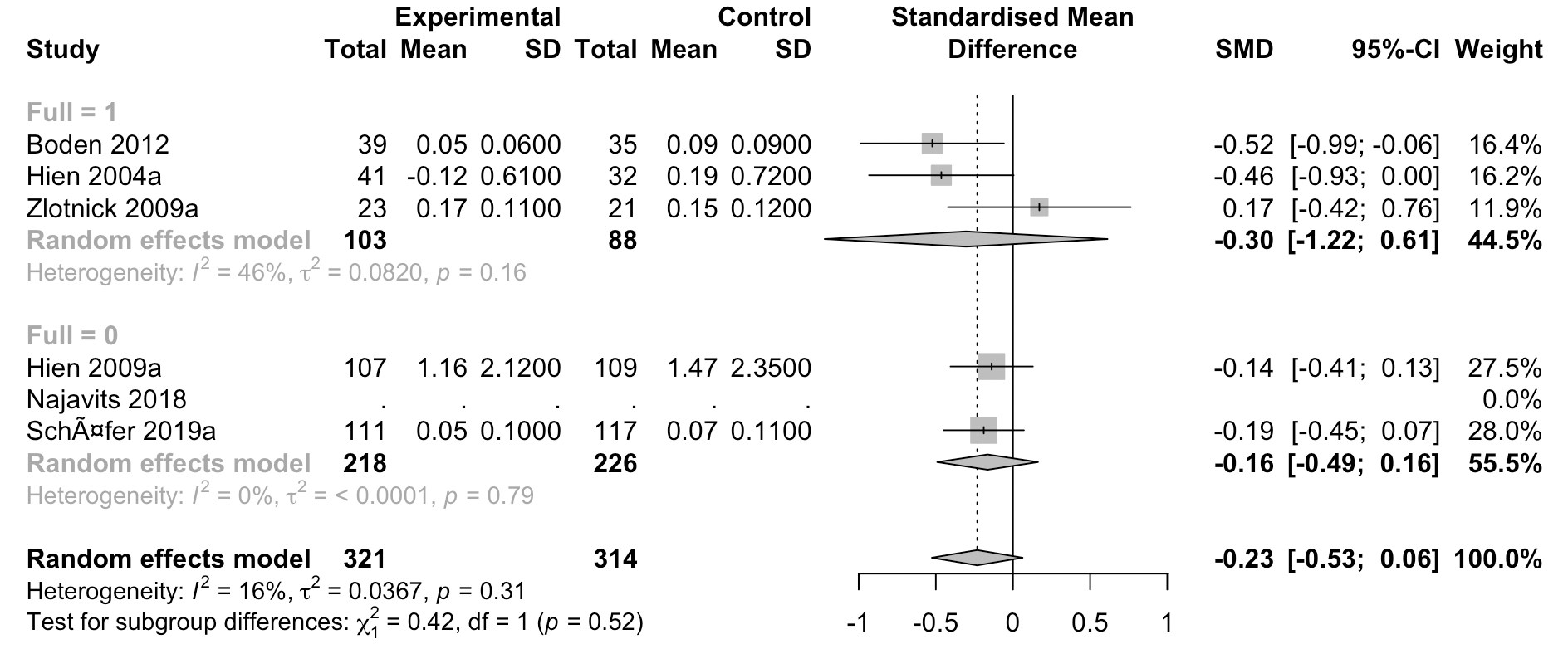 |
| Supplemental Figure 1.14. Meta-analysis results comparing the time effects of Seeking Safety on PTSD measures from baseline to 6 months  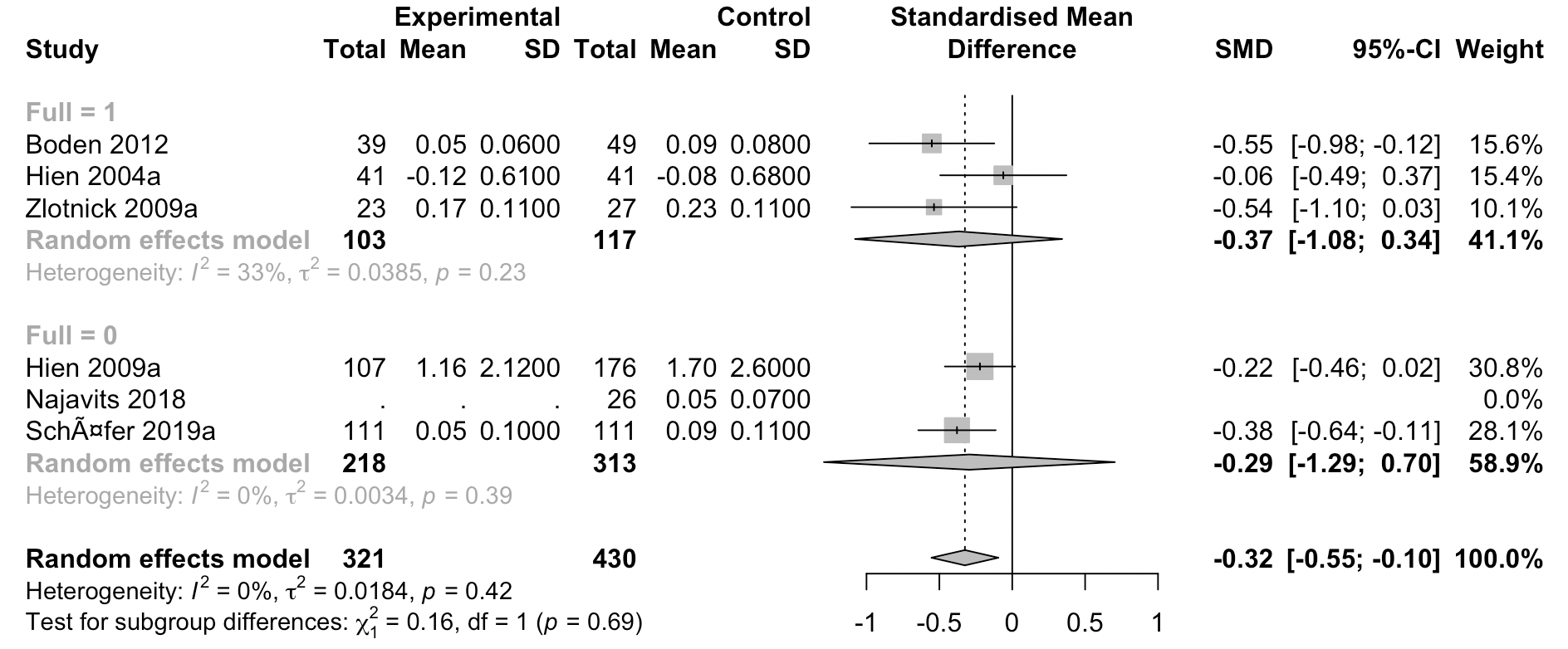 |
| Supplemental Figure 1.15. Meta-analysis results comparing the time by group effects of Seeking Safety on substance use measures from baseline to 6 months  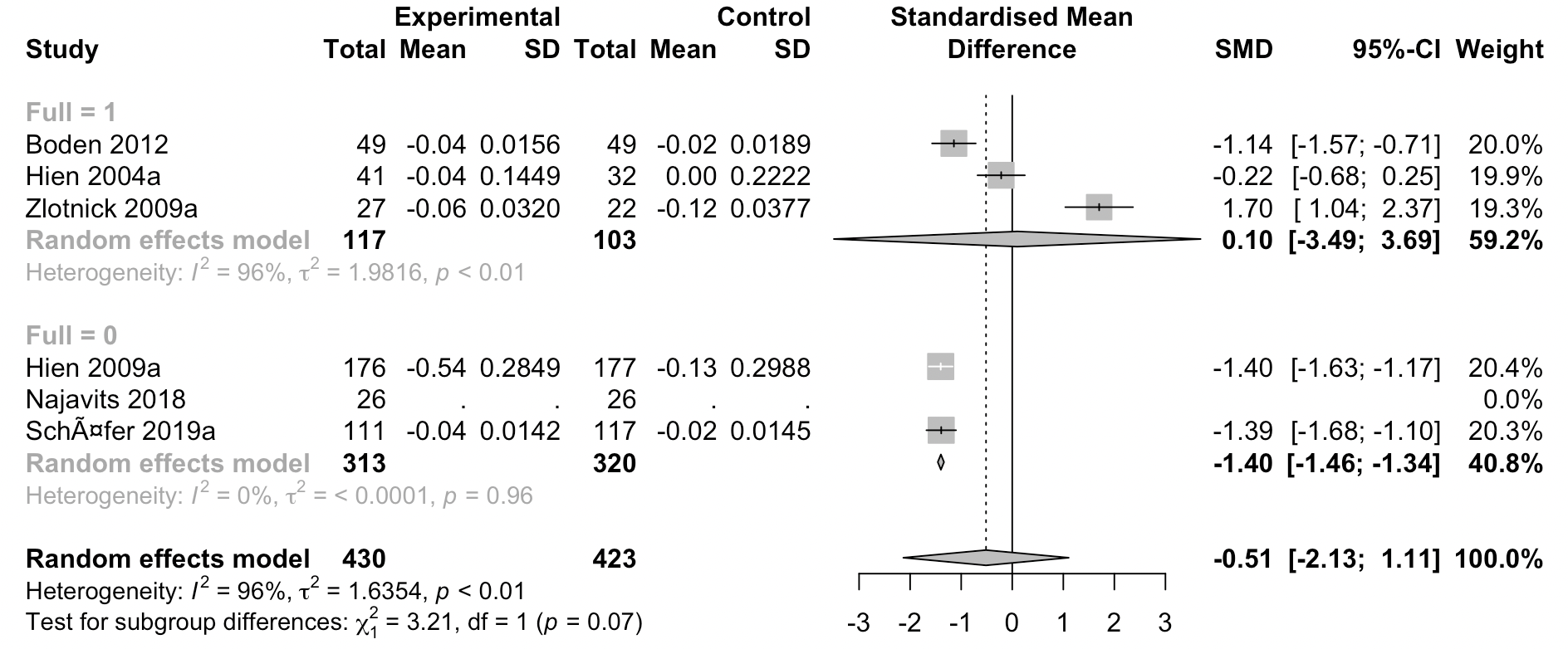 |
| PTSD at 6 months |
| Supplemental Figure 1.16. Meta-analysis results comparing the group effects of Seeking Safety on PTSD measures from baseline to 6 months  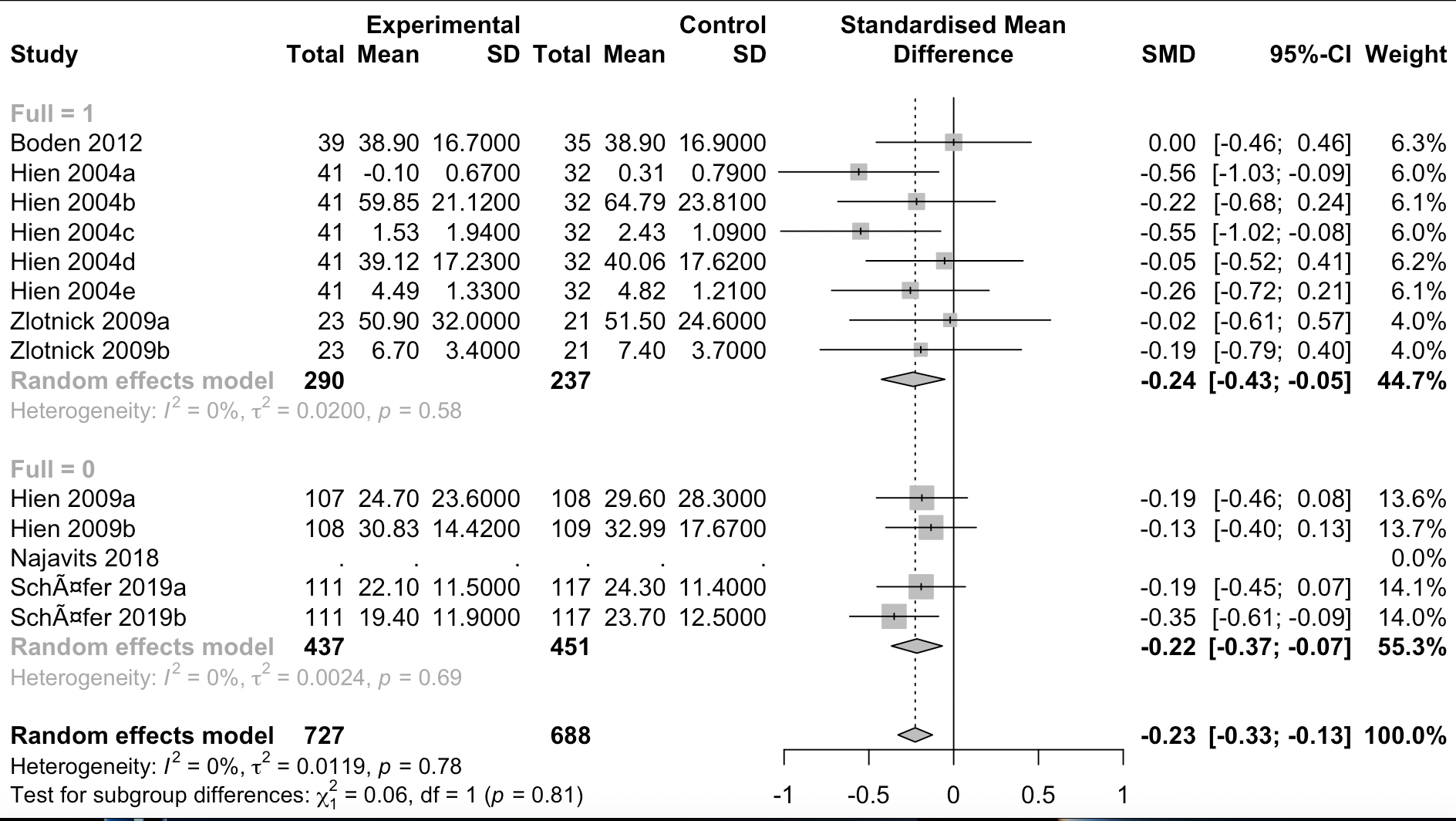 |
| Supplemental Figure 1.17. Meta-analysis results comparing the time effects of Seeking Safety on PTSD measures from baseline to 6 months  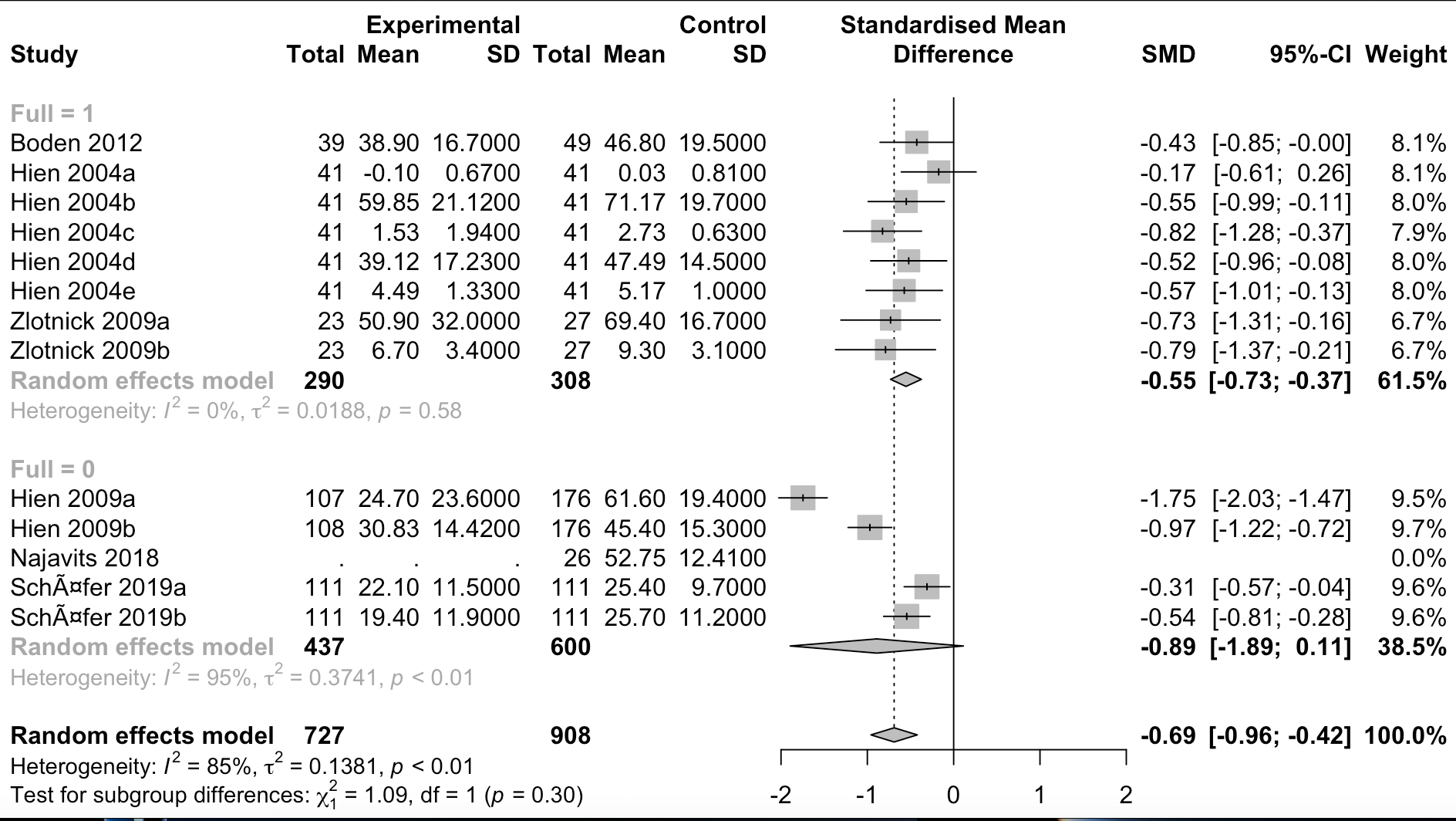 |
| Supplemental Figure 1.18. Meta-analysis results comparing the time by group effects of Seeking Safety on PTSD measures from baseline to 6 months  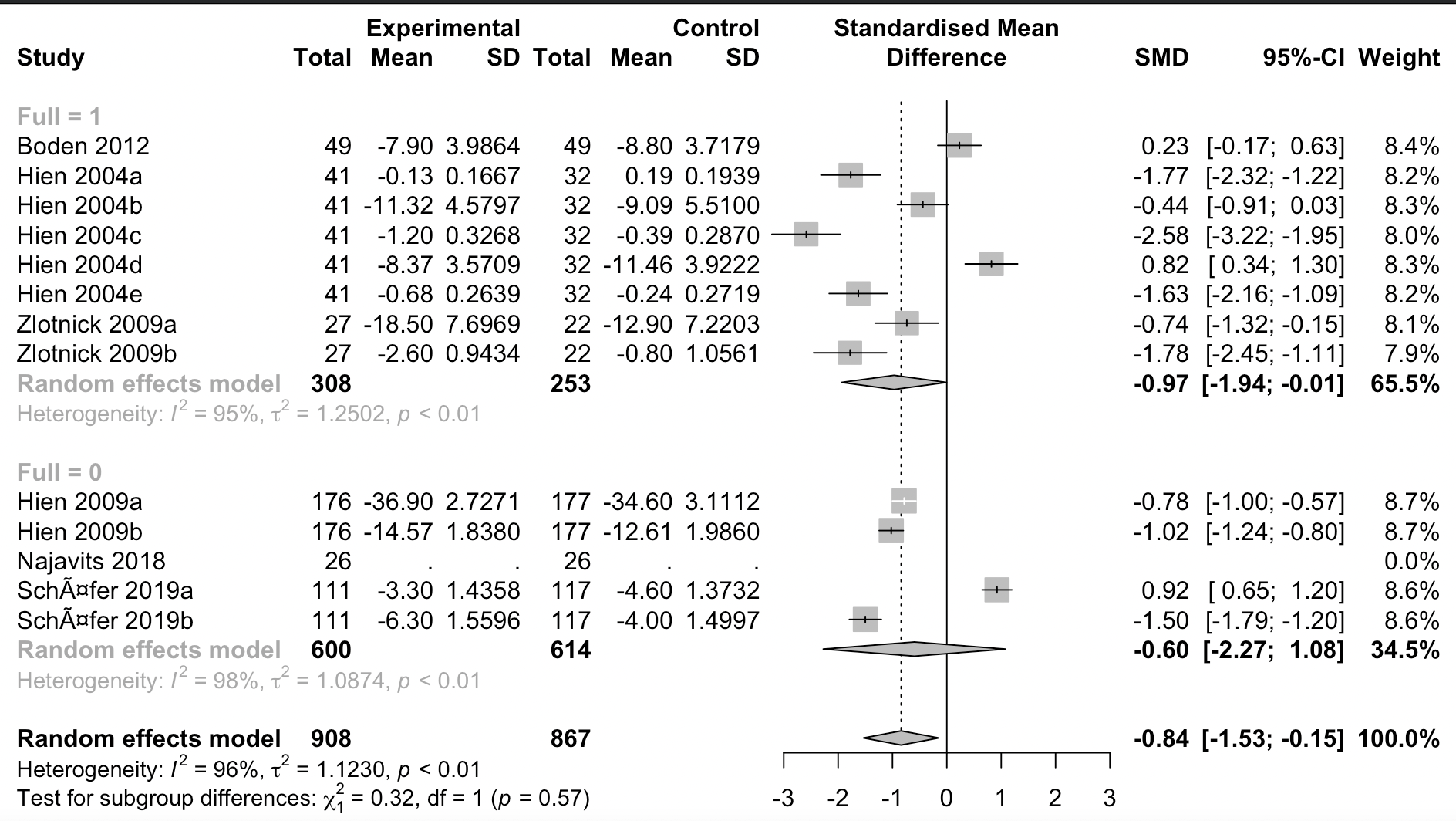 |
| Substance use at 9 months |
| Supplemental Figure 1.19. Meta-analysis results comparing the group effects of Seeking Safety on substance use measures from baseline to 9 months  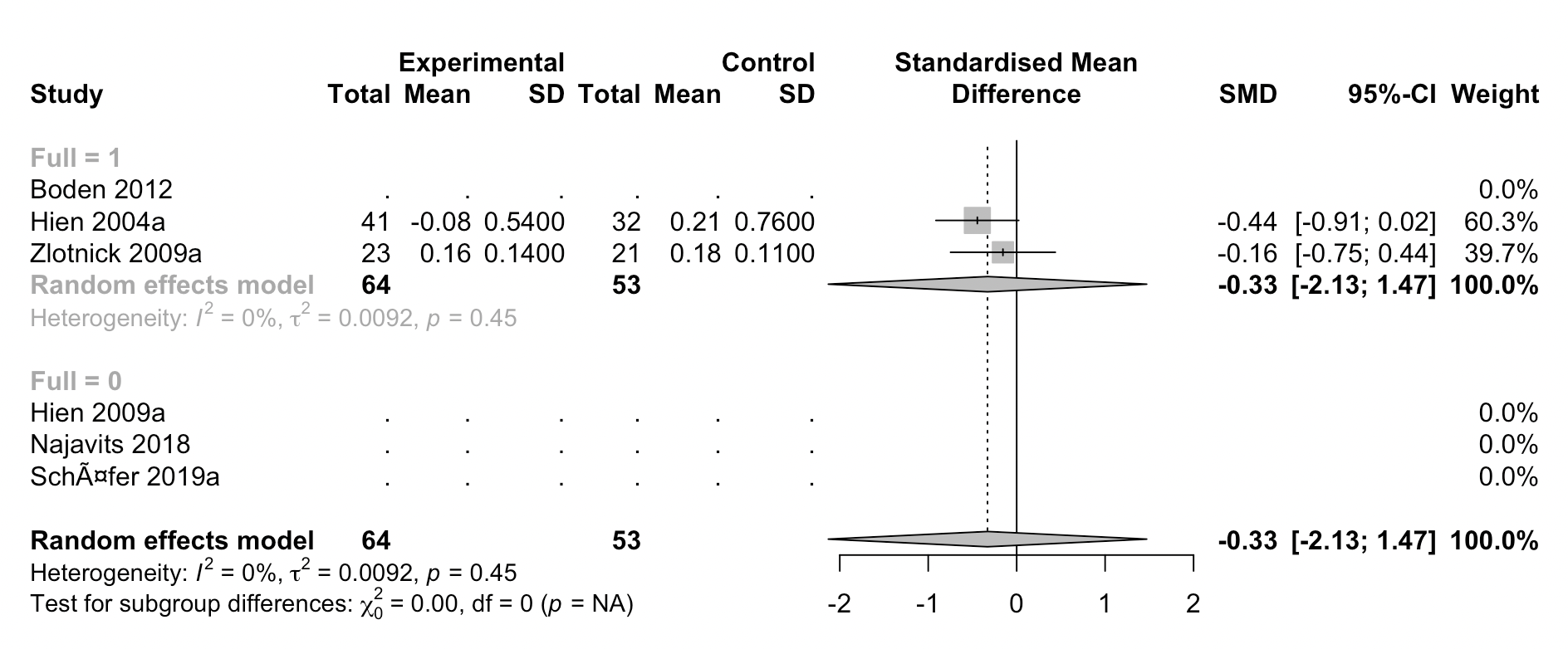 |
| Supplemental Figure 1.20. Meta-analysis results comparing the time effects of Seeking Safety on substance use measures from baseline to 9 months  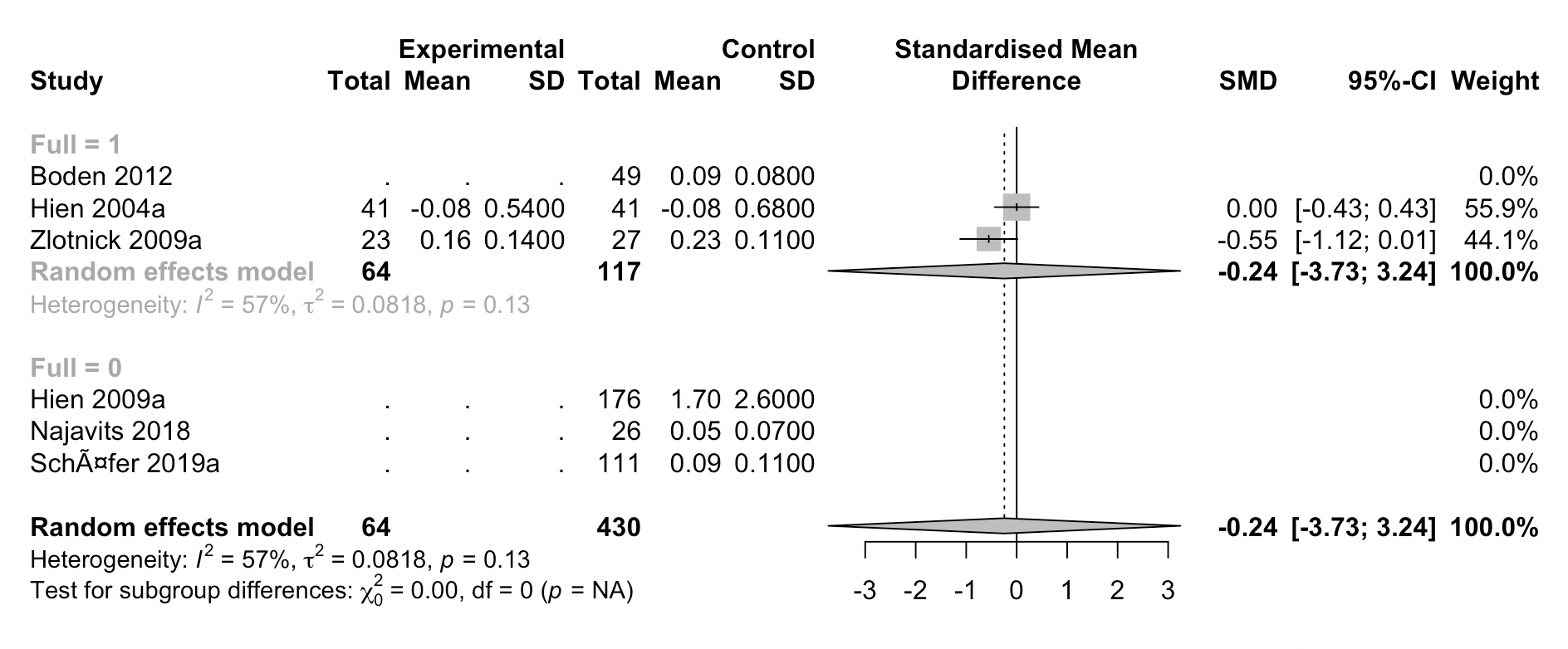 |
| Supplemental Figure 1.21. Meta-analysis results comparing the time by group effects of Seeking Safety on substance use measures from baseline to 9 months  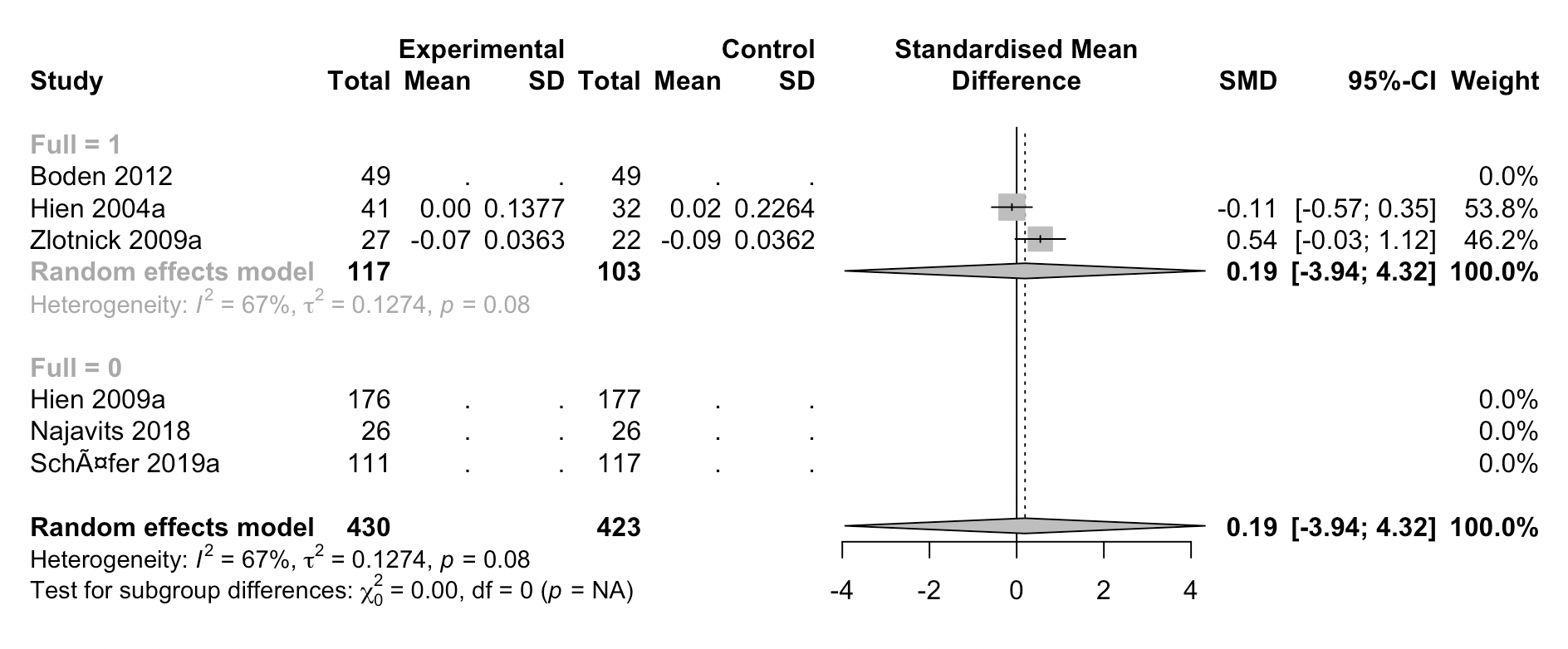 |
| PTSD at 9 months |
| Supplemental Figure 1.22. Meta-analysis results comparing the time by group effects of Seeking Safety on PTSD measures from baseline to 9 months  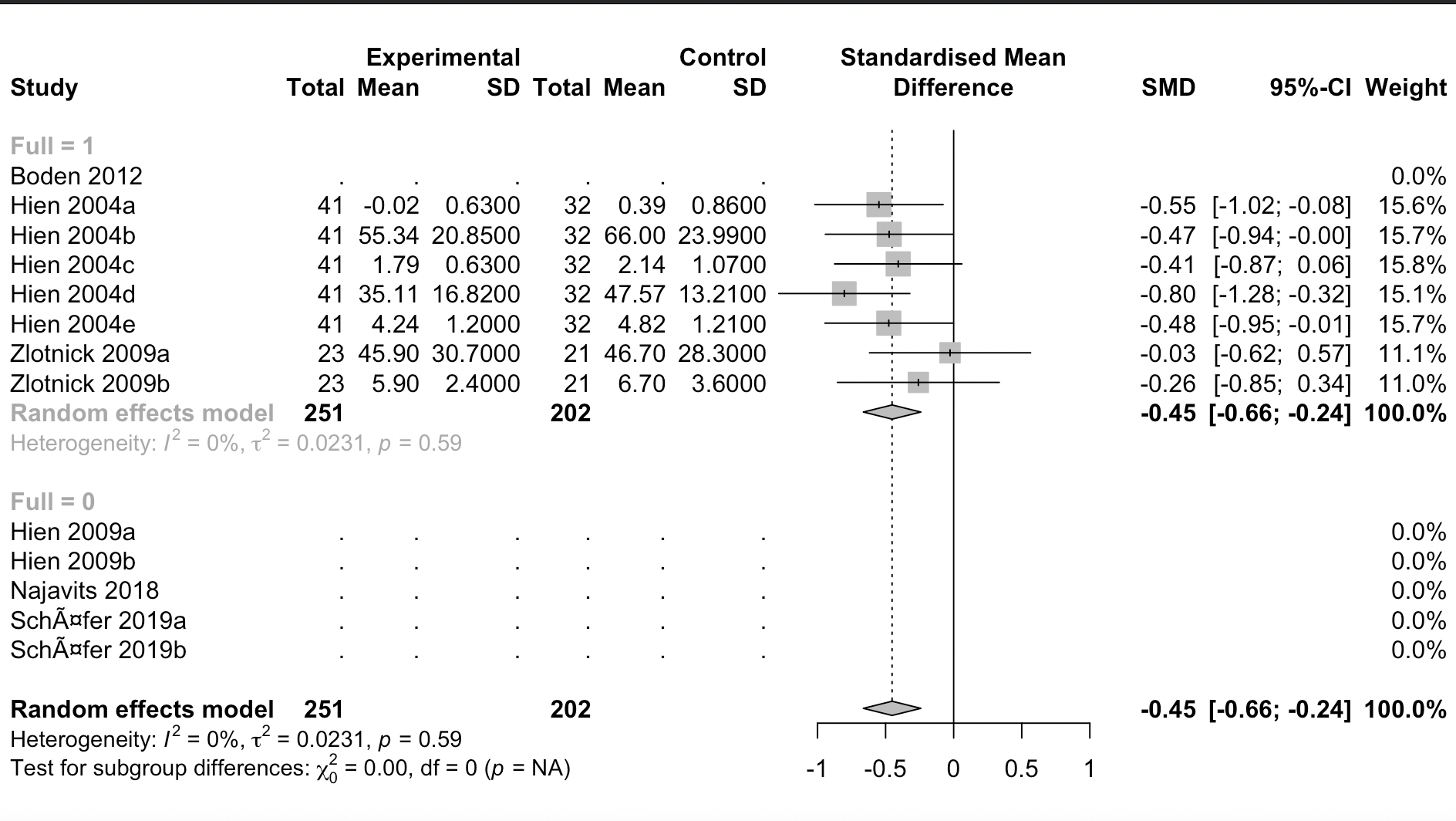 |
| Supplemental Figure 1.23. Meta-analysis results comparing the time effects of Seeking Safety on PTSD measures from baseline to 9 months  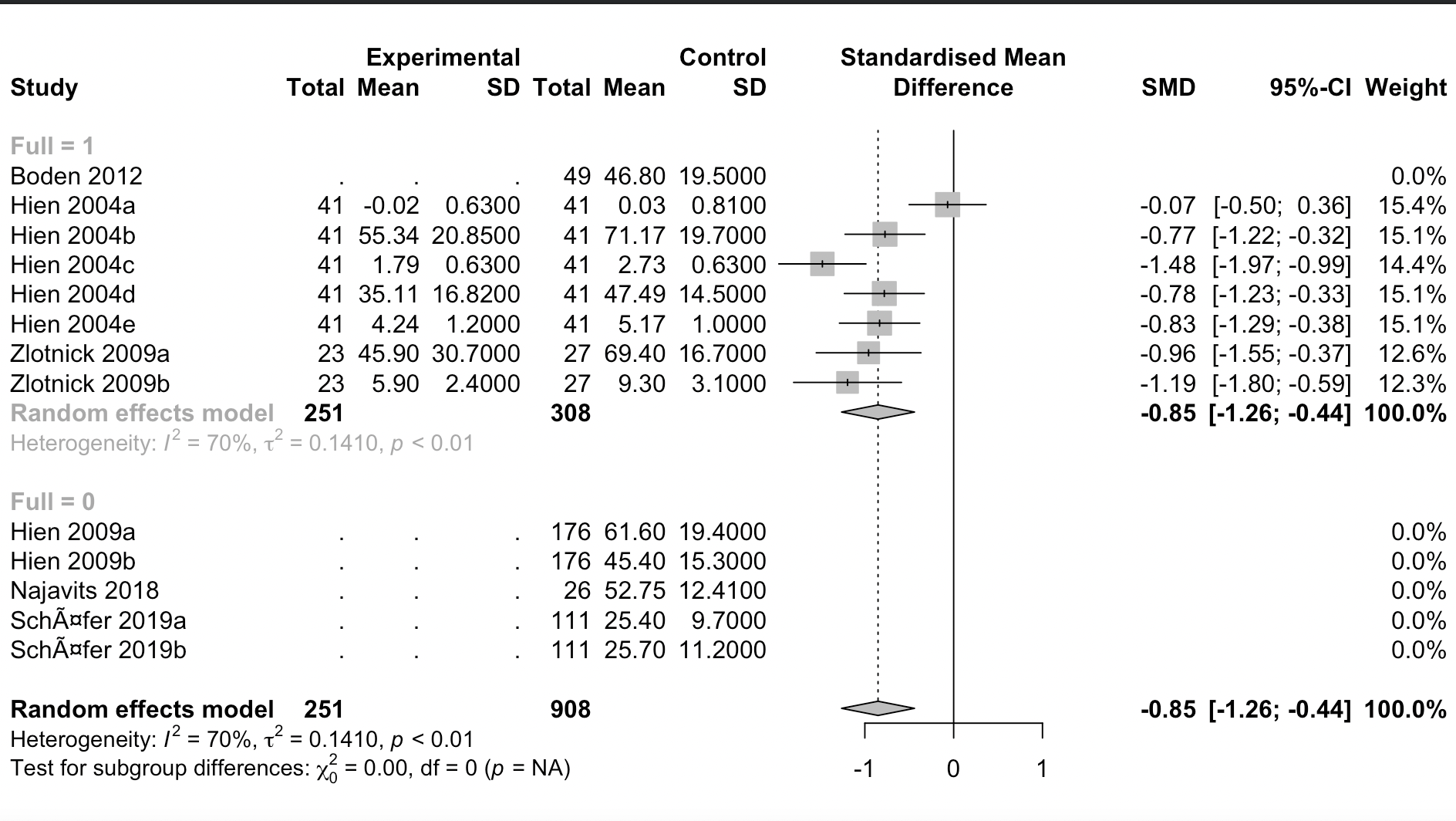 |
| Supplemental Figure 1.24Meta-analysis results comparing the time by group effects of Seeking Safety on PTSD measures from baseline to 9 months  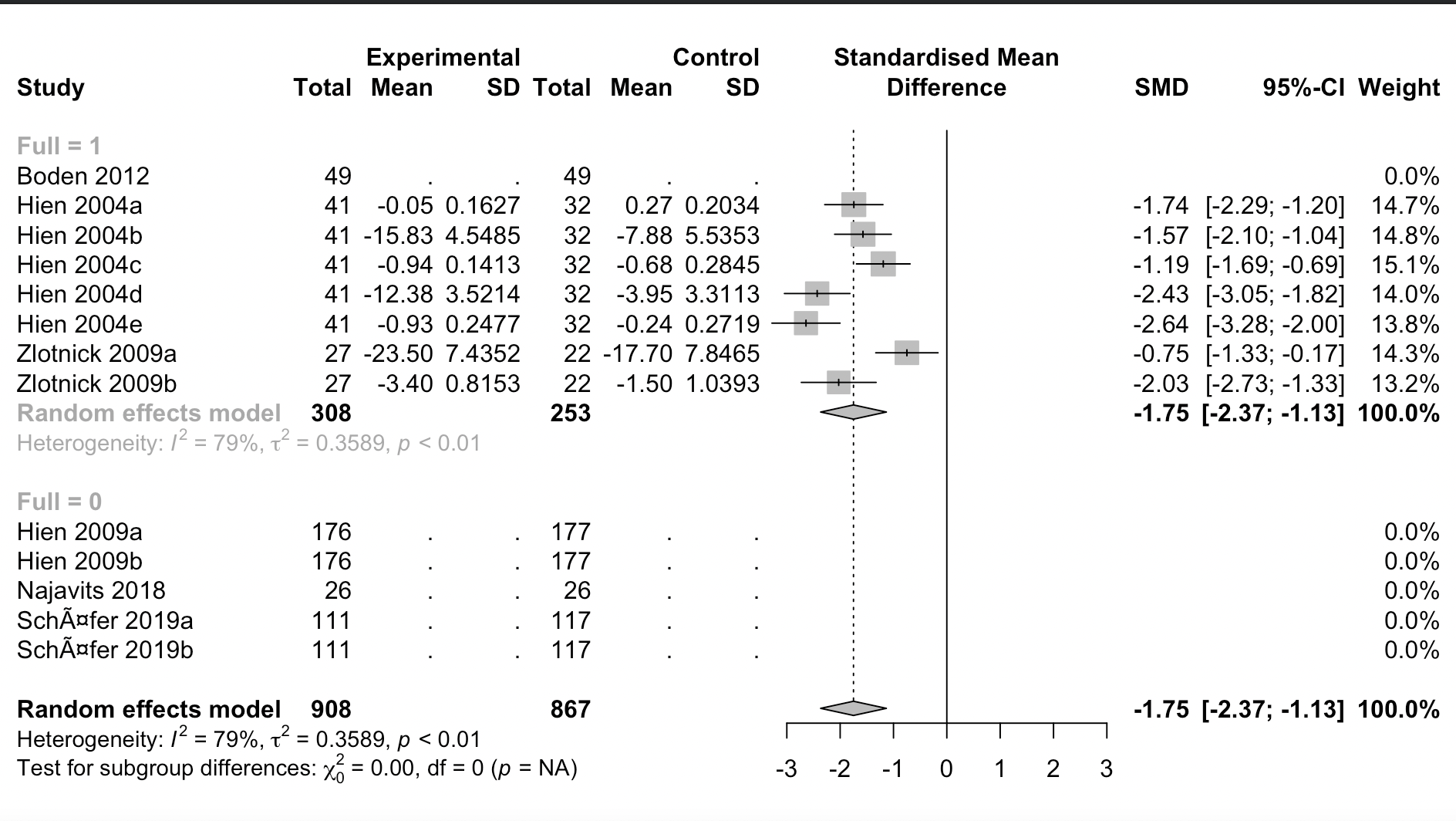 |
